# Synthesis, Biological Evaluation and Docking Studies of Benzoxazoles Derived from Thymoquinone

**DOI:** 10.3390/molecules23123297

**Published:** 2018-12-12

**Authors:** Una Glamočlija, Subhash Padhye, Selma Špirtović-Halilović, Amar Osmanović, Elma Veljović, Sunčica Roca, Irena Novaković, Boris Mandić, Iztok Turel, Jakob Kljun, Snežana Trifunović, Emira Kahrović, Sandra Kraljević Pavelić, Anja Harej, Marko Klobučar, Davorka Završnik

**Affiliations:** 1 Scientific Research Department, Bosnalijek JSC, Jukićeva 53, 71000 Sarajevo, Bosnia and Herzegovina; 2 School of Medicine, University of Mostar, Bijeli Brijeg bb, 88000 Mostar, Bosnia and Herzegovina; 3 Faculty of Pharmacy, University of Sarajevo, Zmaja od Bosne 8, 71000 Sarajevo, Bosnia and Herzegovina; selmaspirtovic@yahoo.com (S.Š.-H.) amar.osmanovic@ffsa.unsa.ba (A.O.); elma.veljovic@ffsa.unsa.ba (E.V.); davorka.zavrsnik@ffsa.unsa.ba (D.Z.); 4 Interdisciplinary Science and Technology Research Academy, University of Pune, 2390-B, Hidayatullah Road, 411001 Pune, India; subhashpadhye@hotmail.com; 5 NMR Centre, Ruđer Bošković Institute, Bijenička cesta 54, 10000 Zagreb, Croatia; Suncica.Roca@irb.hr; 6 ICTM, Center for Chemistry, University of Belgrade, Njegoševa 12, 11000 Belgrade, Serbia; irenan@chem.bg.ac.rs; 7 Faculty of Chemistry, University of Belgrade, Studentski trg 12-16, 11000 Belgrade, Serbia; borism@chem.bg.ac.rs (B.M.); snezanat@chem.bg.ac.rs (S.T.); 8 Faculty of Chemistry and Chemical Technology, University of Ljubljana, Večna pot 113, 1000 Ljubljana, Slovenia; iztok.turel@fkkt.uni-lj.si (I.T.); jakob.kljun@fkkt.uni-lj.si (J.K.); 9 Department of Chemistry, Faculty of Science, University of Sarajevo, Zmaja od Bosne 35, 71000 Sarajevo, Bosnia and Herzegovina; emira_kahrovic@yahoo.com; 10 Centre for High-throughput Technologies, Department of Biotechnology, University of Rijeka, Radmile Matejčić 2, 51000 Rijeka, Croatia; sandrakp@biotech.uniri.hr (S.K.P.); aharej@biotech.uniri.hr (A.H.); mklobucar@biotech.uniri.hr (M.K.)

**Keywords:** thymoquinone, benzoxazoles, anticancer activity, antimicrobial activity, western blotting, molecular docking

## Abstract

Thymoquinone (**TQ**), a natural compound with antimicrobial and antitumor activity, was used as the starting molecule for the preparation of 3-aminothymoquinone (**ATQ**) from which ten novel benzoxazole derivatives were prepared and characterized by elemental analysis, IR spectroscopy, mass spectrometry and NMR (^1^H, ^13^C) spectroscopy in solution. The crystal structure of 4-methyl-2-phenyl-7-isopropyl-1,3-benzoxazole-5-ol (**1a**) has been determined by X-ray diffraction. All compounds were tested for their antibacterial, antifungal and antitumor activities. **TQ** and **ATQ** showed better antibacterial activity against tested Gram-positive and Gram-negative bacterial strains than benzoxazoles. **ATQ** had the most potent antifungal effect against *Candida albicans*, *Saccharomyces cerevisiae* and *Aspergillus brasiliensis*. Three benzoxazole derivatives and **ATQ** showed the highest antitumor activities. The most potent was 2-(4-fluorophenyl)-4-methyl-7-isopropyl-1,3-benzoxazole-5-ol (**1f**). Western blot analyses have shown that this compound inhibited phosphorylation of protein kinase B (Akt) and Insulin-like Growth Factor-1 Receptor (IGF1R β) in HeLa and HepG2 cells. The least toxic compound against normal fibroblast cells, which maintains similar antitumor activities as **TQ**, was 2-(4-chlorophenyl)-4-methyl-7-isopropyl-1,3-benzoxazole-5-ol (**1e**). Docking studies indicated that **1e** and **1f** have significant effects against selected receptors playing important roles in tumour survival.

## 1. Introduction

Thymoquinone (**TQ**) is a natural benzoquinone previously found in *Nigella sativa* L. [1] and some other plant species that are used in traditional medicine for therapeutic treatment of various diseases [2]. **TQ** is a molecule with numerous biological and medicinal activities such as antitumor [3,4,5,6,7], antimicrobial [8,9,10,11], antiepileptic, anti-inflammatory, antioxidant, anti-hypertensive [1], immunomodulatory [11,12], and neuromodulatory effects [13]. The main disadvantage of **TQ** is its low aqueous solubility. There are examples of preparation of **TQ** derivatives with improved solubility and biological effects [14,15,16,17,18].

**TQ** can be used as starting compound for synthesis of benzoxazoles that have aromatic chemical properties and pronounced biological activities. Benzoxazoles can be found in natural sources and there are many examples of synthetic compounds which contain benzoxazole rings [19,20]. Various studies have shown the antitumor activities of benzoxazoles [20,21,22,23,24,25]. Among the main mechanisms is inhibition of topoisomerases I and II, where the benzoxazole ring is crucial for the activity [20]. Other mechanisms are inhibition of cyclooxygenase-2 (COX-2) [26], thymidylate synthase [27] and aurora B kinase [25]. Benzoxazoles have antimicrobial activity [24,28], mainly through binding and depleting metal ions (especially Ca^2+^ and Mg^2+^) from the microorganism cell. Nitrogen in benzoxazole ring is very important for this activity. Moreover, benzoxazoles inhibit enzyme deformylase from *Mycobacterium tuberculosis,* while some benzoxazoles inhibit activity of the human immunodeficiency virus (HIV) [19].

**Figure 1:**
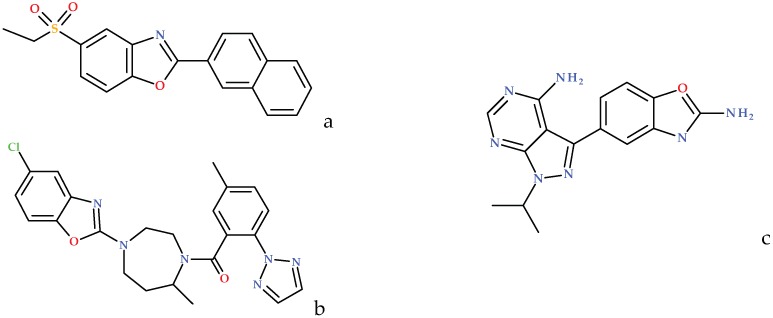
Benzoxazole compounds currently on the market (**a**,**b**) or in clinical trials (**c**). (**a**) Ezutromid is utrophin modulator indicated in Duchenne muscular dystrophy. (**b**) Suvorexant is selective, dual orexin receptor antagonist indicated in insomnia. (**c**) Sapanisertib is an mTOR inhibitor in clinical trials for treatment of different types of cancer.

There are few examples of benzoxazole compounds in clinical trials or on the market (Figure 1).

**Scheme 1:**
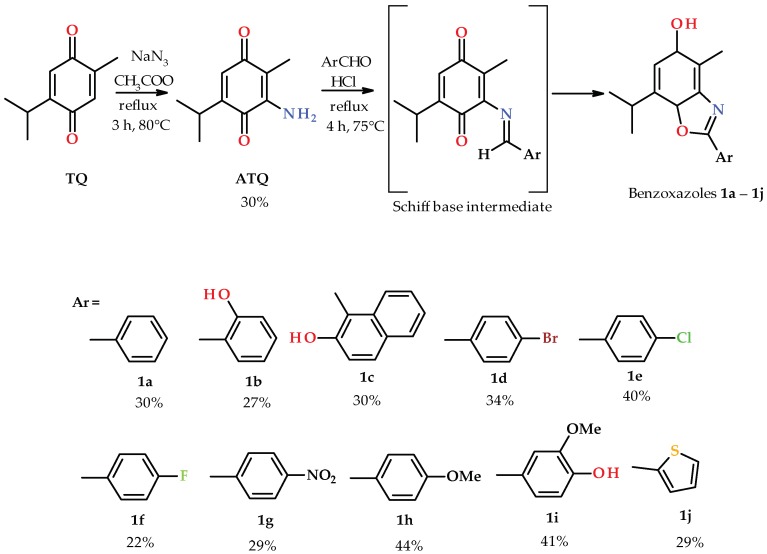
Synthesis of 3-aminothymoquinone (**ATQ**) and series of benzoxazoles **1a**–**1j** from starting compound thymoquinone (**TQ**).

Benzoxazoles are sensitive to hydrolysis which leads to ring opening depending on the structure of molecule, different conditions and pH values [29,30]. Since this process can take place under physiological conditions [29] benzoxazoles can be used as pro-drugs targeted at the specific site of activity [30]. In case of benzoxazole derivatives prepared from **TQ**, the products of hydrolysis would be Schiff bases with expected high biological activity (Scheme 1).

Herein we report the synthesis, antimicrobial and cytotoxic activities and molecular docking studies of **TQ** derivative 3-aminothymoquinone (**ATQ**) and a series of ten novel benzoxazole derivatives produced by reaction between **ATQ** and aromatic aldehydes.

## 2. Results and Discussion

### 2.1. Chemistry

**TQ** was reacted with sodium azide in the presence of catalytic amount of acetic acid to obtain **ATQ**, as previously reported in the literature [31] with slight modifications (Scheme 1). **ATQ** was then reacted with different aryl aldehydes to produce Schiff bases which underwent cyclization into corresponding benzoxazoles (Scheme 1). The reaction itself has been well known for decades, however reports are limited to the synthesis of benzobisoxazoles obtained by reacting two molar equivalents of aldehydes with 2,5-diaminoquinone [32,33], a reaction which was recently employed by the research group of 2016 Nobel laureate Fraser Stoddart in the design of macrocyclic compounds pillar[*n*]arenes [34]. Synthesis of monocyclized products is thus far limited to substructures of extended aromatic systems such as naphthoquinones [35,36], quinolinequinones and indoloquinones [37]. Herein we thus present the novel synthesis of 2-aryl-5-hydroxybenzoxazoles. The structure and purity of the compounds were determined by means of IR and NMR spectroscopy, mass spectrometry, CHN elemental analysis and single crystal X-ray diffraction. Numbering of compounds is presented in Supplementary Scheme S1.

**Figure 2:**
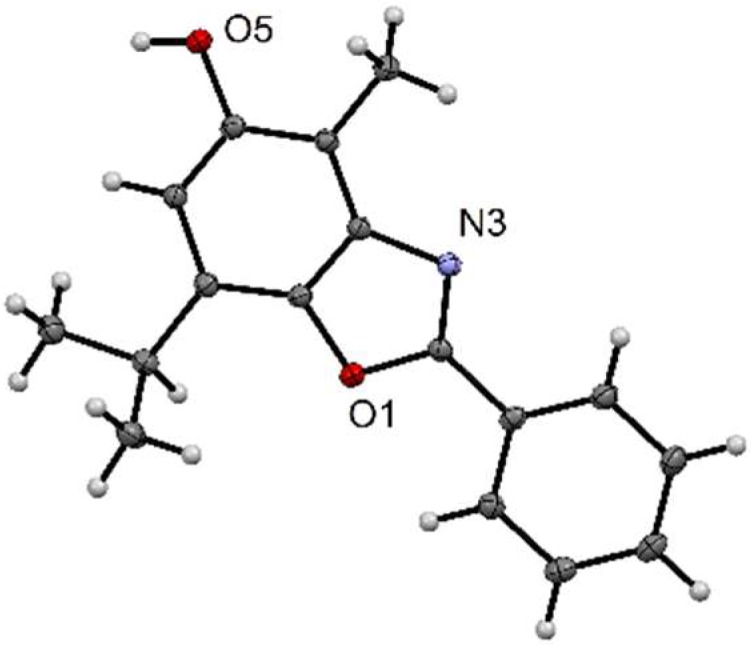
ORTEP view of compound **1a** with heteroatom labelling. Thermal ellipsoids are drawn at 50% probability level.

The crystal structure of representative compound 4-methyl-2-phenyl-7-isopropyl-1,3-benzoxazole-5-ol (**1a**) was determined by single crystal X-ray structure analysis. The ORTEP diagram of compound **1a** is shown in Figure 2. The crystal structure data of compound **1a** are presented in Supplementary Table S1. All analytical data confirmed the assumed structures.

### 2.2. Antibacterial Activity

The compounds **TQ**, **ATQ** and **1a**–**1j** were tested for antibacterial activity against four Gram-positive and four Gram-negative bacteria. All derivatives had lower activity when compared to **TQ** (which showed higher activity than reference drug amikacin) in *Pseudomonas aeruginosa* and *Bacillus subtilis*. The activity was lowered by addition of amino group in position C-3 of **TQ** (**ATQ**) with up to eight-fold higher minimal inhibitory concentration (MIC) values (Table 1).

With the introduction of the benzoxazole core in **1a**–**1j**, antibacterial activity is partially or completely lost.

### 2.3. Antifungal Activity

Antifungal activity of compounds **TQ**, **ATQ** and **1a**–**1j** was tested against three fungi with nystatin as a reference drug. Addition of an amino group in position C-3 of **TQ**, resulted in an improvement of the antifungal activity. **ATQ** showed remarkable inhibitory activity against all tested fungi, with MIC values significantly lower when compared to **TQ** and nystatin (Table 2). With addition of benzoxazole ring, antifungal activity is completely lost and no inhibition zones were seen for compounds **1a**–**1j** in the disk diffusion assay.

### 2.4. Cytotoxicity

MTT assay was used in order to determine IC_50_ values (values at which 50% of cells are inhibited) of compounds (**TQ**, **ATQ**, **1a**–**1j**). Tested compounds have shown various levels of activity against four carcinoma cell lines used (SW620, CFPAC-1, HepG2 and HeLa) (Table 3). Human lung fibroblasts (WI38) were used as healthy control cell line. WI38 is widely used as a model for evaluation of the effects of chemicals on normal cells [38]. It is highly desirable that new drug candidates show no toxic effects in healthy cell lines.

**Figure 3:**
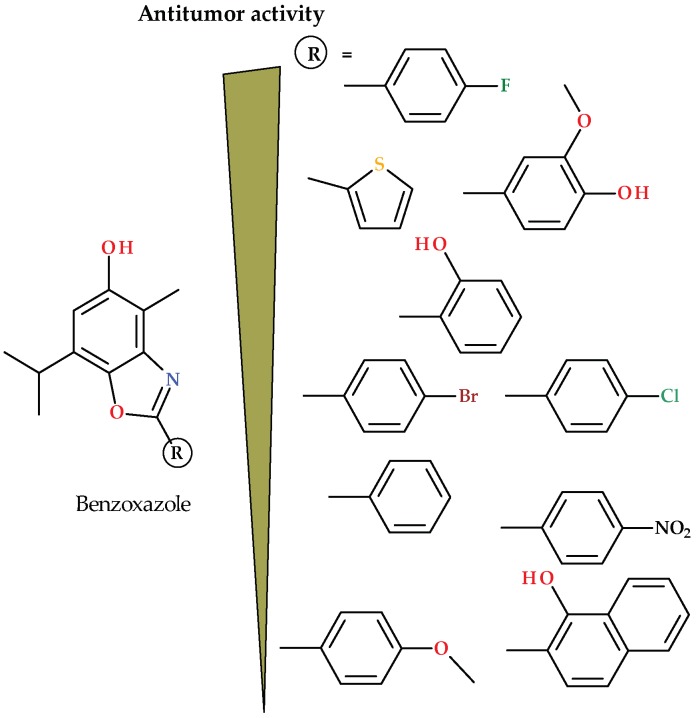
Effects of various substituents on position 2 of benzoxazole ring on antitumor activity of compound.

Different substituents play very important role in the activity of this series of compounds. With addition of amino group at position C-3 of **TQ**, antitumor activity towards all tested cell lines was improved. Addition of benzoxazole ring to the structure, further affects cytotoxic activity, which is depending on substituent at the position 2 of benzoxazole ring (Figure 3). Significantly improved antitumor activity (compared to starting compound **TQ**) had compounds **1f**, **ATQ**, **1i** and **1j**.

Various molecules and signaling pathways play important role in tumor development. Determination of interactions between newly synthesized molecules and cancer signaling pathways is crucial phase in identification and development of new potential drug candidates. Understanding mechanisms of activity directs towards more efficient evaluation of new chemical entities. Western blot technique and molecular docking studies were used for evaluation of the mechanisms involved in the anticancer activity of the most potent compound **1f** and the least toxic derivative **1e**. Results were compared to the ones of starting compound **TQ**.

Among the main mechanisms of **TQ** antitumor activity is inhibition of protein kinase B (Akt) phosphorylation [39,40,41,42] which can be initiated through activation of phosphatase and tensin homolog located on chromosome 10q23 (PTEN) receptor. PTEN is one of the most usually inactivated tumor suppressors with important role in induction of apoptosis and control of cell migration and angiogenesis. It interacts with various signaling pathways in a cell. **TQ** transcriptionally up-regulates PTEN [39,43], which is then leading to apoptosis through several signaling pathways among which is Phosphoinositide 3-kinase (PI3K)/Akt pathway. Decreased levels of phosphorylated Akt were measured after **TQ** treatment in breast [39,41], lymphoma [40], and prostate tumor cells [42].

**Figure 4:**
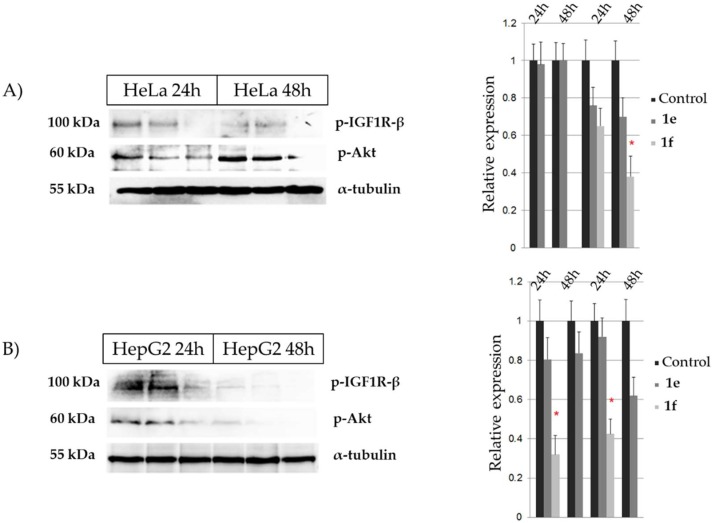
Representative membranes with IGF1R-β and pAkt signals in: (**A**) HeLa cells treated with compounds **1e** and **1f** for 24 h and 48 h. Relative expression is shown on the right panel as the average of three experiments ± standard error. (**B**) HepG2 cells treated with compounds **1e** and **1f** for 24 h and 48 h. Relative expression is shown on the right panel as the average of three experiments ± standard error. Statistically significant changes are marked with an asterisk (*).

In order to determine whether the compounds **1f** and **1e** retain pAkt inhibitory activity of **TQ**, a western blot analysis was performed. Compound **1f** has shown stronger inhibitory activity towards Akt phosphorylation in HepG2 cells where IC_50_ concentration caused significant effect after 24 h and the absence of pAkt signal after 48 h of treatment (Figure 4B). In HeLa cells, significantly decreased pAkt levels were observed only after 48 h treatment (Figure 4A). IC_50_ concentration of compound **1e** showed weak, time and concentration dependent inhibition of Akt phosphorylation in both cancer cell lines (HeLa and HepG2) (Figure 4).

IGF1 Receptor β plays important role in tumor development and metastasis [44,45]. There were no significant changes in IGF1 Receptor β activated form expression upon 24 and 48 h treatments of HeLa and HepG2 cells with compound **1e** (Figure 4). In contrast, IGF1 Receptor β activated form signals were not detectable upon 24 and 48 h treatments of HeLa cells with compound **1f**. Moreover, significantly decreased expression (24 h treatment) and lack of signal (48 h treatment) were observed in compound **1f**-treated HepG2 cells (Figure 4).

### 2.5. Docking Studies

It is already known that compounds containing benzoxazole rings can have different interactions with proteins. The oxygen and nitrogen atoms in the benzoxazole ring can act as hydrogen bond acceptors. The benzoxazole ring has an aromatic planar nature and therefore can form π stacking and π-cation interactions. Because of lipophilic character of benzoxazole derivatives, hydrophobic interactions between benzoxazoles and host proteins can be formed [19].

**Figure 5:**
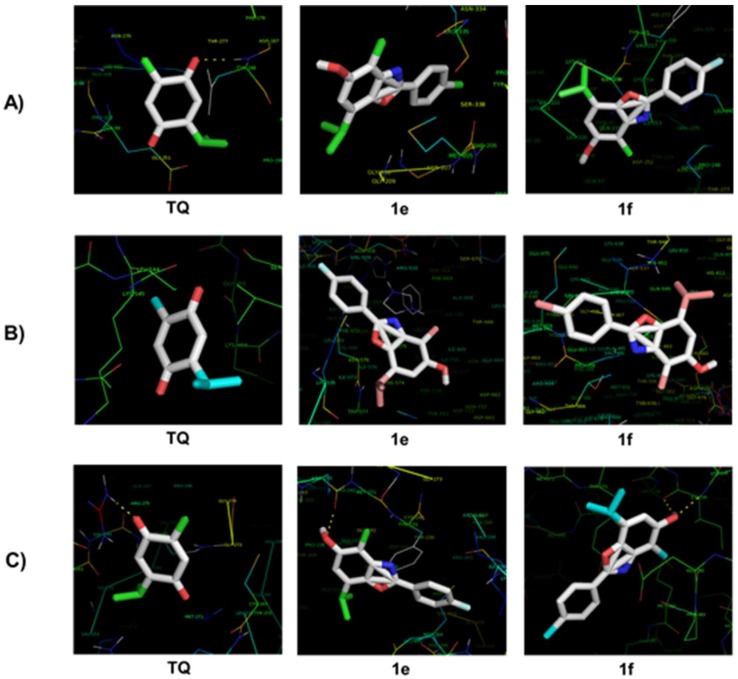
Binding mode of **TQ**, compounds **1e** and **1f** at the active site of the (**A**) PTEN receptor (PDB ID: 1D5R), (**B**) topoisomerase II receptor (PDB ID: 3QX3), and (**C**) NFκB receptor (PDB ID: 1K3Z), as assessed by molecular docking study.

Docking studies of the most potent antitumor compound **1f**, a compound least toxic to normal cells **1e** and a starting compound **TQ**, were performed with the aim to understand the interactions with three molecules crucial in tumorigenesis: PTEN (PDB ID: 1D5R), topoisomerase II (PDB ID: 3QX3) and nuclear factor kappa-light-chain-enhancer of activated B cells (NFκB) (PDB ID: 1K3Z) (Table 4, Figure 5).

The docking studies results indicate that PTEN receptor binding of **TQ** is stronger when compared to **1f** and **1e** compounds. On the other hand, the western blot analyses have revealed that **1f** inhibits Akt phosphorylation significantly, while compound **1e** shows a weak, time and concentration dependent inhibition. Whether those effects are mediated through PTEN remains to be determined.

Topoisomerase II is an enzyme which plays important role in carcinogenesis and it is one of the most studied molecular targets in anticancer research [46,47]. Both **TQ** [48] and benzoxazole derivatives inhibit topoisomerases I and II [20]. Based on docking studies results, and lower binding energies of benzoxazoles, compared to starting compound **TQ**, it can be expected that compounds **1f** and **1e** possess stronger topoisomerase II inhibitory activities than **TQ**.

Inhibition of NFκB is one of the most important characteristics of **TQ** [4,49,50]. NFκB is a transcriptional factor found in cytoplasm which is stimulated by different substances including pro-oxidants, carcinogens and growth factors. After activation, NFκB enters nucleus and binds DNA and stimulates transcription of genes involved in the regulation of apoptosis, proliferation, angiogenesis and inflammation [50]. **TQ** inhibits NFκB through direct interaction with the p65 subunit of NFκB and through suppression of tumor necrosis factor (TNF)-induced IκB kinase (IKK) activation. NFκB activation induced by different carcinogens and inflammatory stimuli is suppressed by **TQ**. As a result, anti-apoptotic and proliferation regulatory proteins are suppressed [50]. According to docking studies results, it can be expected that compounds **1f** and **1e** possess stronger NFκB inhibitory activities than **TQ**.

## 3. Materials and Methods

### 3.1. General Experimental Procedures

All solvents and reagents were used as received from the manufacturers. All chemicals were purchased from Sigma Aldrich (St. Gallen, Switzerland). Melting points were determined on a Kofler Hot Stage Microscope apparatus (Reichert, Wien, Austria) and are uncorrected. The IR spectra were recorded on a Spectrum BX FTIR System (Perkin Elmer, Waltham, MA, USA) as KBr pellets, in the wavelength range from 4500 to 400 cm^−1^. The elemental analysis was accomplished by combustion analysis on a Vario EL III C,H,N,S/O Elemental Analyzer (Elementar, Langenselbold, Hesse, Germany). The NMR spectra in solution were measured in DMSO-*d*_6_ on an AV600 spectrometer (Bruker, Rheinstetten, Germany) at 298 K in 5 mm NMR tubes. ^1^H-NMR spectra were acquired at 600.130 MHz, while ^13^C APT and ^13^C {1H} spectra were acquired at 150.903 MHz. Digital resolutions in ^1^H and ^13^C spectra were 0.37 and 0.60 Hz per point. Chemical shifts (δ/ppm) in ^1^H spectra were referred to the methyl protons of TMS (tetramethylsilane); δ = 0.0 ppm. Chemical shifts (δ/ppm) in ^13^C spectra were referred to the signal of DMSO-*d*_6_; δ = 39.51 ppm. Splitting of peaks in ^1^H-NMR spectra was assigned as: s (singlet), br s (broad singlet), d (doublet), t (triplet), hept (heptet), m (multiplet), dd (doublet of doublets). Double resonance experiments (^1^H-^1^H COSY, ^1^H-^13^C HMQC, ^1^H-^13^C HMBC) were performed in order to assist in signal assignment.

Mass spectral (HRESIMS) data were obtained from an Agilent Technologies 6210 Time-of-Flight LC/MS system consisting of an HPLC instrument Agilent 1200 Series (Agilent Technologies, Waldbronn, Germany), equipped with a degasser, a binary pump, an auto-sampler, a thermostated column compartment and a diode array detector (DAD) and coupled with a 6210 Time-of-Flight LC/MS system (Agilent Technologies, Santa Clara, CA, USA) via an electrospray ionization (ESI) interface. For LC/MS analyses acetonitrile (HPLC grade) purchased from Merck KG (Darmstadt, Germany) and Milli Q water 18.2 MΩcm, obtained from a Millipore Simplicity 185 purification system were used. The mass spectra were recorded in ESI-Mass Spectrum mode (Agilent-6310 ion trap).

X-ray diffraction data were collected on a SuperNova diffractometer (Oxford Diffraction, Abingdon, UK) with a Cu microfocus X-ray source with mirror optics and an Atlas detector. The structures were solved by direct methods implemented in SIR92 [51] and refined by a full-matrix least-squares procedure based on F^2^ using SHELXL-2014 [52]. All non-hydrogen atoms were refined anisotropically. The hydrogen atoms were placed at calculated positions and treated using appropriate riding models. The programs Platon and Mercury were used for data analysis and figure preparation [53,54]. The crystal structure of **1a** has been deposited in the CCDC database and has been assigned the deposition number CCDC 1586630.

### 3.2. General Procedure for the Synthesis of 3-aminothymoquinone (**ATQ**)

**ATQ** was prepared according to the protocol previously reported in literature [31] (Scheme 1) with slight modifications. 1 mmol of **TQ** and 1.3 mmol of sodium azide were dissolved in absolute ethanol. Glacial acetic acid was added as catalyst. Mixture was refluxed for 3 h at 80 °C. Reaction progression was followed by thin layer chromatography (TLC). Reaction was stopped by neutralization with sodium bicarbonate. The obtained crude reaction mixture was filtered and purified by column chromatography with Silicagel 60 as a stationary phase and dichloromethane:diethyl ether:*n*-hexane = 1:2:5 as eluent. The main impurity after synthesis of **ATQ** was **TQ** (*R*_f_ value 0.5–0.6) which was easily separated from **ATQ** (*R*_f_ value 0.38).

*3-Aminothymoquinone* (**ATQ**). Dark red solid (0.054 g, 30%); m.p. 44 °C; IR (KBr) ν_max_ 3462, 3330, 1650, 1580 cm^−1^; ^1^H-NMR (DMSO-*d*_6_) δ 6.43 (2H, s, NH_2_, H-13a, H-13b), 6.29 (1H, s, H-6), 2.87 (1H, hept, *J*_H,H_ = 6.89 Hz, H-9), 1.73 (3H, s, CH3-12), 1.05 (6H, d, *J*_H,H_ = 6.89 Hz, CH3-10, CH3-11); ^13^C-NMR (DMSO-*d*_6_) δ 184.82 (C, C-1), 183.66 (C, C-4), 148.99 (C, C-5), 145.21 (C, C-2), 132.01 (CH, C-6), 106.59 (C, C-3), 25.87 (CH, C-9), 21.11 (CH3, C-10, C-11), 8.65 (CH3, C-12); MS *m*/*z* 180.10 [M + H]^+^ (calcd. forC_10_H_13_NO_2_, 180.1025); anal. C 67.02, H 7.31, N 7.82%, calcd. forC_10_H_13_NO_2_, C 66.78, H 7.58, N 7.71%.

#### General Procedure for the Synthesis of Benzoxazole Derivatives **1a**–**1j**

Aromatic aldehydes were added in equimolar quantities to **ATQ** in absolute ethanol with HCl as catalyst and were refluxed for 4 h at 75 °C to produce Schiff bases which subsequently cyclized into corresponding benzoxazoles. Mixture was poured over crushed ice, precipitate was filtered, washed with distilled water and purified by column chromatography on Silicagel 60. Each compound required different mobile phase for purification (Supplementary Table S2).

*4-Methyl-2-phenyl-7-isopropyl-1,3-benzoxazole-5-ol* (**1a**). White solid (0.081 g, 30.3%); m.p. 164 °C; ^1^H-NMR (DMSO-*d*_6_) δ 9.17 (1H, s, OH), 8.20-8.15 (2H, m, H-12, H-16), 7.63–7.58 (3H, m, H-13, H-14, H-15), 6.76 (1H, s, H-6), 3.26 (1H, hept, *J*_H,H_ = 6.65 Hz, H-8), 2.34 (3H, s, CH3-17), 1.36 (6H, d, *J*_H,H_ = 6.95 Hz, CH3-9, CH3-10); ^13^C-NMR (DMSO-*d*_6_) δ 161.5 (C, C-2), 152.2 (C, C-5), 141.7 (C, C-1), 141.6 (C, C-3), 131.4 (CH, C-14), 129.2 (CH, C-12, C-16), 128.1 (C, C-7), 127.0 (CH, C-13, C-15), 126.9 (C, C-11), 111.6 (C, C-4), 110.6 (CH, C-6), 28.9 (CH, C-8), 22.4 (CH3, C-9, C-10), 10.1 (CH3, C-17); HRESIMS *m*/*z* 268.13339 [M + H]^+^ (calcd. forC_17_H_17_NO_2_, 268.1338); anal. C 76.38, H 6.41, N 5.24%, calcd. forC_17_H_17_NO_2_, C 76.08, H 6.54, N 5.23%.

*2-(2-Hydroxyphenyl)-4-methyl-7-isopropyl-1,3-benzoxazole-5-ol* (**1b**). White solid (0.078 g, 27.5%); m.p. 172 °C; IR (KBr) ν_max_ 3378, 1597, 1545, 1629, 1338, 1155 cm^−1^; ^1^H-NMR (DMSO-*d*_6_, 600.130 MHz) δ 11.42 (1H, s, OH-12), 9.35 (1H, s, OH-5), 8.01 (1H, d, *J*_H,H_ = 7.76 Hz, H-16), 7.75 (1H, t, *J*_H,H_ = 8.36 Hz, H-14), 7.11 (1H, d, *J*_H,H_ = 8.21 Hz, H-13), 7.08 (1H, t, *J*_H,H_ = 7.63 Hz, H-15), 6.81 (1H, s, H-6), 3.28 (1H, hept, *J*_H,H_ = 6.98 Hz, H-8), 2.34 (3H, s, CH3-17), 1.36 (6H, d, *J*_H,H_ = 6.84 Hz, CH3-9, CH3-10); ^13^C-NMR (DMSO-*d*_6_) δ 161.7 (C, C-2), 157.5 (CH, C-12), 152.6 (C, C-5), 140.0 (C, C-1), 139.5 (C, C-3), 133.5 (CH, C-14), 128.4 (C, C-7), 127.0 (CH, C-16), 119.9 (CH, C-15), 117.0 (CH, C-13), 110.92 (C, C-4), 110.87 (CH, C-6), 110.5 (C, C-11), 28.8 (CH, C-8), 22.4 (CH3, C-9, C-10), 10.0 (CH3, C-17); HRESIMS *m*/*z* 284.1 [M + H]^+^ (calcd. forC_17_H_17_NO_3_, 284.1287); anal. C 67.76, H 6.35, N 4.65%, calcd. forC_17_H_17_NO_3_^.^H_2_O, C 67.55, H 6.41, N 4.54%.

*2-(2-Hydroxynaphtalene)-4-methyl-7-isopropyl-1,3-benzoxazole-5-ol* (**1c**). White solid (0.100 g, 30%); m.p. 204 °C; IR (KBr) ν_max_ 3338, 1576, 1624, 1338, 1134 cm^−1^; ^1^H-NMR (DMSO-*d*_6_) δ 12.64 (1H, s, OH-22), 9.33 (1H, s, OH-25), 8.64 (1H, d, *J*_H,H_ = 8.63 Hz, H-17), 8.07 (1H, d, *J*_H,H_ = 8.63 Hz, H-14), 7.96 (1H, d, *J*_H,H_ = 8.63 Hz, H-20), 7.65 (1H, t, *J*_H,H_ = 7.84 Hz, H-18), 7.45 (1H, t, *J*_H,H_ = 7.06 Hz, H-19), 7.35 (1H, t, *J*_H,H_ = 7.84 Hz, H-13), 6.83 (1H, s, H-6), 3.32 (1H, hept, *J*_H,H_ = 6.90 Hz, H-8), 2.38 (3H, s, CH3-21), 1.40 (6H, d, *J*_H,H_ = 6.95, Hz, CH3-9, CH3-10); ^13^C-NMR (DMSO-*d*_6_) δ 162.3 (C, C-2), 158.6 (C, C-12), 152.6 (C, C-5), 140.4 (C, C-1), 138.9 (C, C-3), 134.1 (CH, C-14), 130.9 (C, C-16), 129.0 (CH, C-20), 128.3 (CH, C-18), 128.0 (CH, C-7), 127.96 (C, C-15), 123.7 (CH, C-19), 123.6 (CH, C-17), 118.9 (CH, C-13), 110.7 (CH, C-6), 103.7 (C, C-11), 29.1 (CH, C-8), 22.3 (CH3, C-9, C-10), 10.1 (CH3, C-21), *n.d.* (C, C-4); HRESIMS *m*/*z* 334.14366 [M+H]^+^ (calcd. forC_21_H_19_NO_3_, 334.1443); anal. C 75.66, H 5.74, N 4.20%, calcd. forC_21_H_19_NO_3_, C 75.18, H 5.77, N 4.17%.

*2-(4-Bromophenyl)-4-methyl-7-isopropyl-1,3-benzoxazole-5-ol* (**1d**). White solid (0.090 g, 33.7%); m.p. 190 °C; ^1^H-NMR (DMSO-*d*_6_) δ 9.21 (1H, s, OH), 8.10 (2H, d, *J*_H,H_ = 8.70 Hz, H-13, H-15), 7.80 (2H, d, *J*_H,H_ = 8.70 Hz, H-12, H-16), 6.78 (1H, s, H-6), 3.25 (1H, hept, *J*_H,H_ = 5.99 Hz, H-8), 2.33 (3H, s, CH3-17), 1.34 (6H, d, *J*_H,H_ = 6.67 Hz, CH3-9, CH3-10); ^13^C-NMR (DMSO-*d*_6_) δ 160.7 (C, C-2), 152.3 (C, C-5), 141.7 (C, C-3), 141.6 (C, C-1), 132.3 (CH, C-13, C-15), 128.8 (CH, C-12, C-16), 128.2 (C, C-7), 126.1 (C, C-11), 125.0 (C, C-14), 111.7 (C, C-4), 110.9 (C, C-6), 28.8 (CH, C-8), 22.4 (CH3, C-9, C-10), 10.1 (CH3, C-17); HRESIMS *m*/*z* 346.04319 [M + H]^+^ (calcd. forC_17_H_16_BrNO_2_, 346.0443); anal. C 58.97, H 4.66, N 4.05%, calcd. forC_17_H_16_BrNO_2_, C 58.36, H 4.58, N 3.99%.

*2-(4-Chlorophenyl)-4-methyl-7-isopropyl-1,3-benzoxazole-5-ol* (**1e**). Yellow solid (0.120 g, 39.8%); m.p. 180 °C; ^1^H-NMR (DMSO-*d*_6_) δ 9.23 (1H, s, OH), 8.17 (2H, d, *J*_H,H_ = 8.52 Hz, H-12, H-16), 7.66 (2H, d, *J*_H,H_ = 8.52 Hz, H-13, H-15), 6.78 (1H, s, H-6), 3.25 (1H, hept, *J*_H,H_ = 6.65 Hz, H-8), 2.34 (3H, s, CH3-17), 1.35 (6H, d, *J*_H,H_ = 6.95 Hz, CH3-9, CH3-10); ^13^C-NMR (DMSO-*d*_6_) δ 160.6 (C, C-2), 152.3 (C, C-5), 141.7 (C, C-3), 141.6 (C, C-1), 136.1 (C, C-14), 129.4 (CH, C-12, C-16), 128.7 (CH, C-13, C-15), 128.2 (C, C-7), 125.8 (C, C-11), 111.6 (C, C-4), 110.8 (CH, C-6), 28.8 (CH, C-8), 22.4 (CH3, C-9, C-10), 10.1 (CH3, C-17); HRESIMS *m*/*z* 302.09382 [M + H]^+^ (calcd. forC_17_H_16_ClNO_2_, 302.0948); anal. C 67.66, H 5.34, N 4.64%, calcd. forC_17_H_16_ClNO_2_, C 67.34, H, 5.29, N 4.59%.

*2-(4-Fluorophenyl)-4-methyl-7-isopropyl-1,3-benzoxazole-5-ol* (**1f**). White solid (0.060 g, 22.4%); m.p. 152 °C; ^1^H-NMR (DMSO-*d*_6_) δ 9.18 (1H, s, O*H*), 8.22 (2H, dd, *J*_H,H_ = 8.78 Hz, *J*_H,F_ = 5.50 Hz, H-12, H-16), 7.43 (2H, dd, *J*_H,H_ = 8.78, Hz, *J*_H,F_ = 8.78 Hz, H-13, H-15), 6.76 (1H, s, H-6), 3.25 (1H, hept, *J*_H,H_ = 6.65 Hz, H-8), 2.34 (3H, s, CH3-17), 1.34 (6H, d, *J*_H,H_ = 6.95 Hz, CH3-9, CH3-10); ^13^C-NMR (DMSO-*d*_6_) δ 163.9 (C, *J*_C,F_ = 256.0 Hz, C-14), 160.7 (C, C-2), 152.2 (C, C-5), 141.68 (C, C-1), 141.66 (C, C-3), 129.5 (CH, *J*_C,F_ = 9.13 Hz, C-12, C-16), 128.2 (C, C-7), 123.6 (C, C-11), 116.4 (CH, *J*_C,F_ = 22.4 Hz, C-13, C-15), 111.6 (C, C-4), 110.6 (CH, C-6), 28.8 (CH, C-8), 22.4 (CH3, C-9, C-10), 10.1 (CH3, C-17); HRESIMS *m*/*z* 286.12359 [M + H]^+^ (calcd. forC_17_H_16_FNO_2_, 286.1243); anal. C 71.56, H 5.65, N 4.91%, calcd. forC_17_H_16_FNO_2_, C 71.12, H 5.42, N 4.90%.

*4-Methyl-2-(4-nitrophenyl)-7-isopropyl-1,3-benzoxazole-5-ol* (**1g**). Yellow solid (0.090 g, 28.8%); m.p. 188 °C; ^1^H-NMR (DMSO-*d*_6_) δ 9.29 (1H, s, OH), 8.42–8.40 (4H, m, H-12, H-13, H-15, H-16), 6.83 (1H, s, H-6), 3.28 (1H, hept, *J*_H,H_ = 6.91 Hz, H-8), 2.36 (3H, s, CH3-17), 1.36 (6H, d, *J*_H,H_ = 7.14 Hz, CH3-9, CH3-10); ^13^C-NMR (DMSO-*d*_6_) δ 159.6 (C, C-2), 152.5 (C, C-5), 148.7 (C, C-14), 142.1 (C, C-1), 141.7 (C, C-3), 132.4 (C, C-11), 128.5 (C, C-7), 128.1 (CH, C-12, C-16), 124.4 (CH, C-13, C-15), 112.0 (C, C-4), 111.8 (CH, C-6), 28.8 (HC, C-8), 22.4 (CH3, C-9, C-10), 10.0 (CH3, C-17); HRESIMS *m*/*z* 313.11768 [M + H]^+^ (calcd. forC_17_H_16_N_2_O_4_, 313.1188); anal. C 65.38, H 5.16, N 8.97%, calcd. forC_17_H_16_N_2_O_4_, C 64.73, H 4.87, N 8.89%.

*2-(4-Methoxyphenyl)-4-methyl-7-isopropyl-1,3-benzoxazole-5-ol***(1h**). White solid (0.130 g, 43.7%); m.p. 178 °C; ^1^H-NMR (DMSO-*d*_6_) δ 9.11 (1H, s, OH), 8.11 (2H, d, *J*_H,H_ = 7.98 Hz, H-12, H-16), 7.14 (2H, d, *J*_H,H_ = 7.98 Hz, H-13, H-15), 6.71 (1H, s, H-6), 3.86 (3H, s, OCH3), 3.24 (1H, hept, *J*_H,H_ = 6.65 Hz, H-8), 2.32 (3H, s, CH3-17), 1.34 (6H, d, *J*_H,H_ = 6.95 Hz, CH3-9, CH3-10); ^13^C-NMR (DMSO-*d*_6_) δ 161.74 (C, C-14), 161.67 (C, C-2), 152.1 (C, C-5), 141.8 (C, C-3), 141.4 (C, C-1), 128.7 (CH, C-12, C-16), 127.9 (C, C-7), 119.4 (C, C-11), 114.6 (CH, C-13, C-15), 111.3 (C, C-4), 110.0 (CH, C-6), 55.4 (CH3, C-19), 28.8 (CH, C-8), 22.5 (CH3, C-9, C-10), 10.1 (CH3, C-17); HRESIMS *m*/*z* 298.14311 [M + H]^+^ (calcd. forC_18_H_19_NO_3_, 298.1443); anal. C 72.71, H 6.44, N 4.71%, calcd. forC_18_H_19_NO_3_, C 71.96, H 6.43, N 4.62%.

*2-(4-Hydroxy-3-methoxyphenyl)-4-methyl-7-isopropyl-1,3-benzoxazole-5-ol* (**1i**). White solid (0.130 g, 41.5%); m.p. 168 °C; ^1^H-NMR (DMSO-*d*_6_) δ 9.82 (1H, s, OH-18), 9.07 (1H, s, OH-23), 7.65–7.61 (2H, m, H-12, H-16), 6.96 (1H, d, *J*_H,H_ = 8.48 Hz, H-15), 6.69 (1H, s, H-6), 3.90 (3H, s, OCH3), 3.24 (1H, hept, *J*_H,H_ = 6.98 Hz, H-8), 2.32 (3H, s, CH3-17), 1.33 (6H, d, *J*_H,H_ = 6.89 Hz, CH3-9, CH3-10); ^13^C-NMR (DMSO-*d*_6_) δ 162.0 (C, C-2), 152.0 (C, C-5), 150.0 (C, C-14), 147.9 (C, C-13), 141.9 (C, C-1), 141.4 (C, C-3), 127.8 (C, C-7), 120.8 (CH, C-16), 118.1 (C, C-11), 115.9 (CH, C-15), 111.2 (C, C-4), 110.4 (CH, C-12), 109.7 (CH, C-6), 55.7 (CH3, C-20), 28.7 (CH, C-8), 22.5 (CH3, C-9, C-10), 10.1 (CH3, C-17); HRESIMS *m*/*z* 314.13840 [M + H]^+^ (calcd. forC_18_H_19_NO_4_, 314.1392); anal. C 68.99, H 6.11, N 4.47%, calcd. forC_18_H_19_NO_4_, C 68.38, H 6.08, N 4.48%.

*4-Methyl-7-isopropyl-2-(thiophene-2-yl)-1,3-benzoxazole-5-ol* (**1j**). White solid (0.080 g, 29.3%); m.p. 152 °C; IR (KBr) ν_max_ 3108, 1568, 1302, 1115, 856, 1036, 720 cm^−1^; ^1^H-NMR (DMSO-*d*_6_) δ 9.18 (1H, s, OH), 7.90 (2H, d, *J*_H,H_ = 4.17 Hz, H-13, H-15), 7.28 (1H, t, *J*_H,H_ = 4.64 Hz, H-14), 6.74 (1H, s, H-6), 3.25 (1H, hept, *J*_H,H_ = 6.65 Hz, H-8), 2.30 (3H, s, CH3-16), 1.33 (6H, d, *J*_H,H_ = 7.01, Hz, CH3-9, CH3-10); ^13^C-NMR (DMSO-*d*_6_) δ 157.7 (C, C-2), 152.3 (C, C-5), 141.6 (C, C-3), 141.2 (C, C-1), 131.1 (CH, C-15), 129.6 (CH, C-13), 129.2 (C, C-11), 128.7 (CH, C-14), 128.0 (C, C-7), 111.4 (C, C-4), 110.4 (CH, C-6), 28.7 (CH, C-8), 22.4 (CH3, C-9, C-10), 10.1 (CH3, C-16); HRESIMS *m*/*z* 274.08914 [M + H]^+^ (calcd. forC_15_H_15_NO_2_S, 274.0902); anal. C 65.91, H 5.53, N 5.12, S 11.73%, calcd. forC_15_H_15_NO_2_S, C 65.55, H 5.54, N 5.07, S 11.46%.

### 3.3. Biological Assays

#### 3.3.1. Antimicrobial Activity

##### Compounds and Microorganisms

Antimicrobial activity of compounds was evaluated in vitro on eight bacterial strains (*Escherichia coli* ATCC 25922 (ATCC = American Type Culture Collection, Manassas, VA, USA), *Pseudomonas aeruginosa* ATCC 9027, *Proteus hauseri* ATCC 13315, *Klebsiella pneumoniae* ATCC 10031, *Staphylococcus aureus* ATCC 6538, *Bacillus subtilis* ATCC 6633, *Clostridium sporogenes* ATCC 19404 and *Micrococcus luteus* ATCC 10240) and three fungi (*Candida albicans* ATCC 10231, *Saccharomyces cerevisiae* ATCC 9763 and *Aspergillus brasiliensis* ATCC 16404).

##### Diffusion and Micro-Dilution Assays

Agar well diffusion and micro-dilution assays were used for evaluation of antimicrobial activity. To each sterile Petri dish (90 mm diameter) 22 mL of nutrient agar and 100 μL of bacterial suspension (10^6^ cell per dish) or 22 mL Sabouraud dextrose agar and 100 μL of fungi (10^5^ spores per dish) were added. A well with the diameter of 8 mm was punched using a sterile cork borer. The tested compounds were dissolved in DMSO at the concentration 10 mg mL^−1^ and 100 μL of this solution was added to each well. Final concentration of tested compounds was 1 mg/well. At the same time, amikacin and nystatin in final concentration 30 μg/well were used as reference drugs for antibacterial and antifungal testing, respectively. Petri dishes were maintained at room temperature for 4 h, and after that they were incubated: bacterial strains 24 h at 37 °C, and fungi 48 h at 25 °C. After incubation, inhibition zone diameters were measured by using a Readbiotic Microbiological Culture Analyzer (International PBI SpA, Milan, Italy), with the precision of 0.1 mm. Experiments were performed in triplicate, and average values presented.

Micro-dilution assay was used for determination of tested compounds MIC. A serial dilution method in sterile 96-well microtitre plates was performed. Fresh Mueller–Hinton broth (for bacteria) and Sabouraud dextrose broth (for fungi) were used. In sterile physiological solution, suspensions containing 1.5 × 10^8^ CFU mL^−1^ for bacteria and 1.5 × 10^7^ CFU mL^−1^ for fungi were made (according to McFarland). One hundred microliters of broth were added to each well of the plate. Stock solutions of tested compounds were made in DMSO at concentration 10 mg mL^−1^, and then 100 μL of the compound stock solution was added to each well of the first column. Double dilution was made in whole row for one tested compound, and repeated for all tested compounds in other rows. Amikacin served as positive control for bacteria, while nystatin served as positive control for fungi. The solvent (DMSO) was used as the negative control. Afterwards, each well was inoculated with 10 μL of microorganism suspension. After incubation, 24 h at 37 °C for bacteria and 48 h at 25 °C for fungi, results were read. The lowest concentration where no growth of microorganism was detected represented MIC.

#### 3.3.2. Cytotoxic Activity

##### Compounds and Cell Lines

The tested compounds were dissolved in DMSO in five, 10-fold dilutions (0.01–100 µM). The cell lines HeLa (cervical carcinoma), SW620 (colorectal adenocarcinoma, metastatic), HepG2 (hepatocellular carcinoma), CFPAC-1 (ductal pancreatic adenocarcinoma), and WI38 (human lung fibroblast cell line), were cultured as monolayers and maintained in Dulbecco’s modified Eagle medium (DMEM) supplemented with 10% fetal bovine serum (FBS), 2 mM L-glutamine, 100 U mL^−1^ penicillin and 100 μg mL^−1^ streptomycin in a humidified atmosphere with 5% CO_2_ at 37 °C.

##### MTT Assay

The MTT assay was used for evaluation of effects of tested compounds on viability of carcinoma and control cell lines. About 5000 cells per well were seeded onto a series of standard 96-well plates on day 0. Test agents were then added and incubated for further 72 h. Working dilutions of tested compounds were freshly prepared in the growth medium, on the day of testing. The solvent (DMSO) was also tested for eventual inhibitory activity by adjusting its concentration to be the same as in the working concentrations (DMSO concentration never exceeded 0.1%). After 72 h of incubation, the cell growth rate was evaluated by performing the MTT assay: experimentally determined absorbance values were transformed into a cell percentage growth (PG) using the formulas proposed by NIH and described previously [55]. This method directly relies on control cells number at the day of assay because it compares the growth of treated cells with the growth of untreated cells in control wells on the same plate, the results are therefore a percentile difference from the calculated expected value.

The IC_50_ values for each compound were calculated from dose-response curves using linear regression analysis by fitting the mean test concentrations that give PG values above and below the reference value. If, however, all of the tested concentrations produce PGs exceeding the respective reference level of effect (e.g., PG value of 50) for a given cell line, the highest tested concentration is assigned as the default value (in the screening data report that default value is preceded by a “>” sign). Each test point was performed in quadruplicate in three individual experiments. The results were statistically analyzed (Analysis of variance (ANOVA), Tukey post-hoc test at *p* < 0.05). Finally, the effects of the tested substances were evaluated by plotting the mean percentage growth for each cell type in comparison to control on dose response graphs.

##### Western Blot

HeLa and HepG2 cells were seeded in six well plate, at 400,000 cells/well, and treated with compounds **1e** and **1f** at micromolar concentrations assessed as active on the cell cycle without immediate cell death activity, compound **1e** (HeLa IC_50_: 42.67 µM and HepG2 IC_50_: 39.48 µM) and compound **1f** (HeLa IC_50_: 4.13 µM and HepG2 IC_50_: 9.36 µM) for 24 h and 48 h. Protein lysates were lysed in the buffer 50 mM Tris HCl (pH 8), 150 mM NaCl, 1% NP-40, 0.5% sodium deoxycholate, 0.1% SDS and protease and phosphatase inhibitor cocktail (Roche, Basel, Switzerland). A total of 50 μg of proteins were resolved on 10% SDS polyacrylamide gels by use of Mini-protean cell (Bio-Rad, Hercules, CA, USA). Membranes were incubated with primary antibodies against phospho (Tyr 1135) -IGF1 Receptor β (1:1000, rabbit mAb, Cell Signalling Technology, Danvers, MA, USA), phospho (Ser 473) Akt (1:1000, rabbit mAb, Cell Signalling Technology) and α tubulin (1:1000, mouse mAb, Sigma Aldrich) at 4 °C overnight. Secondary antibody linked to anti-mouse was used at 1:1000 (Dako, Santa Clara, CA, USA) and to anti-rabbit at 1:1000 (Dako). Signals were visualized by Western Lightening Chemiluminescence Reagent Plus Kit (Perkin Elmer, USA) on the ImageQuant LAS500 (GE Healthcare, Chicago, IL, USA). Signal intensities of particular bands were normalized with the intensity of the loading control α tubulin and compared in Quantity One software (Bio-Rad). The values are expressed as average value of three independent experiments ± standard error of the mean. Statistical analysis was performed in Microsoft Excel (Microsoft Corporation, Redmond, WA, USA) by using the ANOVA (*p* < 0.05).

### 3.4. Docking Studies

The docking studies were performed with the aim of determination of probable antitumor activity mechanisms of the most potent **1f** and the least toxic **1e** compounds in comparison with starting compound **TQ**. 3D coordinates for the crystal structures of PTEN (PDB ID: 1D5R), topoisomerase II (PDB ID: 3QX3) and NFκB (PDB ID: 1K3Z) were obtained from Brookhaven protein data bank [56,57].

AutoDockTools (ADT, The Scripps Research Institute, San Diego, CA, USA) software was used for preparation and analysis of docking simulations. Lamarckian genetic algorithm (LGA) was used for searching conformers which are energetically the most favorable. Up to 100 conformers of each compound were analyzed. Coordination x, y, z network with dimensions 126 × 126 × 126 dots, and distance between dots 0.375 Å, was prepared and centered into main catalytic part of the receptor. Size of population was 150, with the random selection of individuals. Maximal number of energetic calculations was 2,500,000, maximal number of generated conformations was 27,000, with number of individuals restricted to 1, mutation speed 0.02, recombination speed 0.8, 2 Å translation, quaternion 50° and torsion angle 50°. 100 LGA calculations were performed, with cluster tolerance 2 Å, external network energy 1000, maximal initial energy 0 and maximal number of repetitions 10,000. Structures of tested compounds were optimized in Chem3D Ultra 9.0.1. software (Perkin Elmer) with the use of AM1 semi empirical method [58]. Docking studies were performed by using AutoDock 4.0. software (The Scripps Research Institute). Discovery Studio Visualizer software (Biovia, San Diego, CA, USA) was used for preparation of receptors and ligands. PyMol 1.1 (Schrödinger, LLC, New York, NY, USA) was used for final visualization of the best conformers (tested compound-receptor).

## 4. Conclusions

**TQ** was used as a starting compound for preparation of **ATQ** and benzoxazoles **1a**–**1j** which were studied for their antimicrobial and cytotoxic activities. **ATQ** had stronger antifungal activity than **TQ** and reference drug nystatin, while benzoxazoles **1a**–**1j** didn’t show antifungal activity. All prepared derivatives had weaker antibacterial activity in comparison to **TQ**. Amination at position C-3 of **TQ**, resulted in increased toxicity against four selected cancer cell lines. An additional increase in cytotoxicity was gained through synthesis of benzoxazole derivatives with specific substituents at the 2 position of the benzoxazole ring. The most potent antitumor compound **1f** contains the *p*-fluoro-phenyl group, while the derivative **1e**, which is the least toxic towards human lung fibroblasts, contains a *p*-chlorophenyl substituent at the 2 position of the benzoxazole ring. Moreover, benzoxazole derivatives **1i** (containing 4-hydroxy-3-methoxyphenyl group at the 2 position of the benzoxazole ring) and **1j** (containing a thiophene-2-yl group at the 2 position of the benzoxazole ring) had stronger antitumor activities, when compared to **TQ**. The compound **1f** inhibited phosphorylation of Akt and IGF1 Receptor β in HeLa and HepG2 cell lines, while the compound **1e** showed weak, time and concentration dependent inhibition of Akt phosphorylation in both cancer cell lines. According to docking studies results, it can be expected that compounds **1f** and **1e** have significant effects towards topoisomerase II, NFκB and PTEN receptors. With the aim of better understanding the mode of action of newly synthesized compounds, their interactions with molecules involved in tumor cells signaling need to be further evaluated.

## Figures and Tables

**Table 1:** In vitro antibacterial activity represented as minimum inhibitory concentration (MIC/μM) values of investigated compounds.

Bacteria	Compound		
	TQ	ATQ	Amikacin
	MIC (μM)		
*E. coli*	29.84	111.59	8.54
*P. aeruginosa*	59.68	217.61	85.38
*P. hauseri*	29.84	217.61	11.95
*K. pneumoniae*	59.68	435.22	13.71
*S. aureus*	29.84	111.59	18.78
*B. subtilis*	59.68	217.61	71.72
*C. sporogenes*	121.80	435.22	25.61
*M. luteus*	59.68	59.68	3.42

**Table 2:** In vitro antifungal activity presented as minimum inhibitory concentration (MIC/mM) values of investigated compounds.

Fungus	Compound		
	TQ	ATQ	Nystatin
	MIC (mM)		
*C. albicans*	3.81	1.75	2.70
*S. cerevisiae*	1.90	0.22	1.35
*A. braziliensis*	3.81	0.88	1.35

**Table 3:** IC_50_ values (values at which 50% of cells are inhibited) (µM) of compounds **TQ**, **ATQ**, and **1a**–**1j** in tested cell lines. The most potent compound in each tumour cell line is bolded. The least toxic compound against WI38 is bolded. SW620, CFPAC, HepG2 and HeLa are carcinoma cell lines, WI38 is healthy control human fibroblast cell line.

Compound	Cell Line				
	SW620	CFPAC	HepG2	HeLa	WI38
	IC_50_ (µM)				
**TQ**	39.95	48.26	23.97	28.85	19.83
**ATQ**	25.82	30.87	8.26	15.74	9.41
**1a**	>100	90.22	39.34	32.22	19.89
**1b**	43.68	31.02	32.27	25.00	29.81
**1c**	>100	>100	>100	>100	84.48
**1d**	51.53	34.51	30.46	30.10	57.61
**1e**	79.88	49.47	39.48	42.67	**>100**
**1f**	**5.82**	33.81	9.36	**4.13**	5.54
**1g**	54.93	>100	>100	>100	6.21
**1h**	>100	97.00	>100	>100	75.89
**1i**	55.30	40.58	**4.58**	6.38	9.32
**1j**	31.55	**27.01**	7.28	10.27	9.97

**Table 4:** Binding characteristics of **TQ**, **1e** and **1f** to phosphatase and tensin homolog located on chromosome 10q23 (PTEN) (PDB ID: 1D5R), topoisomerase II (PDB ID: 3QX3) and nuclear factor kappa-light-chain-enhancer of activated B cells (NFκB) (PDB ID: 1K3Z) receptors, as assessed by molecular docking study.

Compound	PTEN			Topoisomerase II			NFκB		
	Binding Energy (kcal/mol)	Ki * Value (mM)	No. of H bonds	Binding Energy (kcal/mol)	Ki * Value (mM)	No. of H bonds	Binding Energy (kcal/mol)	Ki * Value (mM)	No. of H bonds
**TQ**	−4.02	1.12	1	−3.02	6.1	0	−4.75	0.328	1
**1e**	−3.57	2.41	0	−4.32	0.677	0	−6.02	0.039	2
**1f**	−3.88	1.43	0	−3.80	1.65	0	−5.58	0.082	1

* Ki = inhibition constant.

## Supplementary Materials

The following are available online. Scheme S1: Numbering scheme for 3-aminothymoquinone (**ATQ**) and series of benzoxazoles (**1a**–**1j**); Table S1: Mobile phases used in purification of compounds **1a**–**1j**; Table S2: The crystal structure data of compound **1a**; IR spectra for compounds **ATQ**, **1b**, **1c** and **1j**; Mass spectra for compounds **ATQ** and **1a**–**1j**; ^1^H- and ^13^C-NMR spectra for compounds **ATQ** and **1a**–**1j**.
