# Supplementary material for: Synthesis, Biological Evaluation and Docking Studies of Benzoxazoles Derived from Thymoquinone
Source: Molecules. 2018 Dec 12;23(12):3297. doi: 10.3390/molecules23123297 (PMC6321291; doi:10.3390/molecules23123297)
Supplement: Supplementary file 1 [file molecules-23-03297-s001.zip › molecules-399050- supplementary/molecules-399050-IR spectra.pdf]

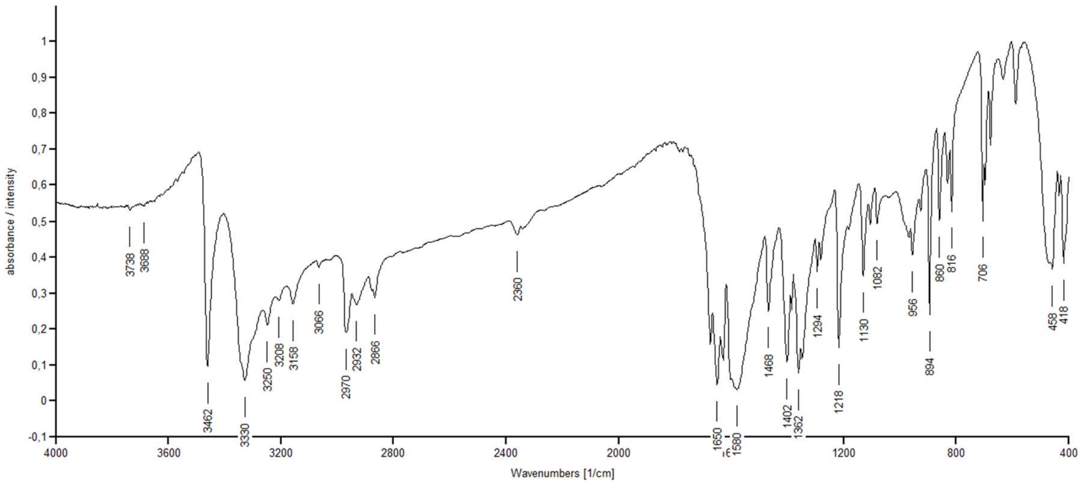

IR spectra for compound **ATQ**

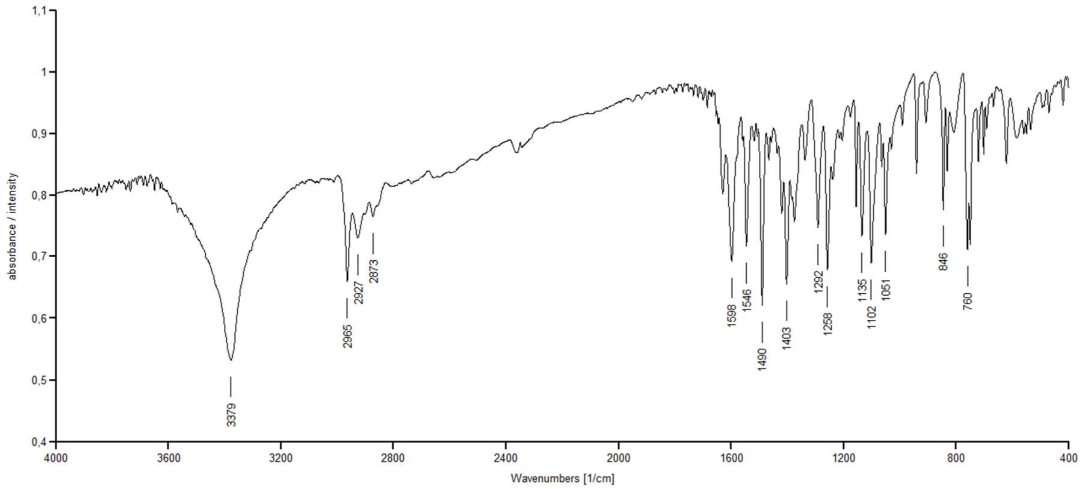

IR spectra for compound **1b**

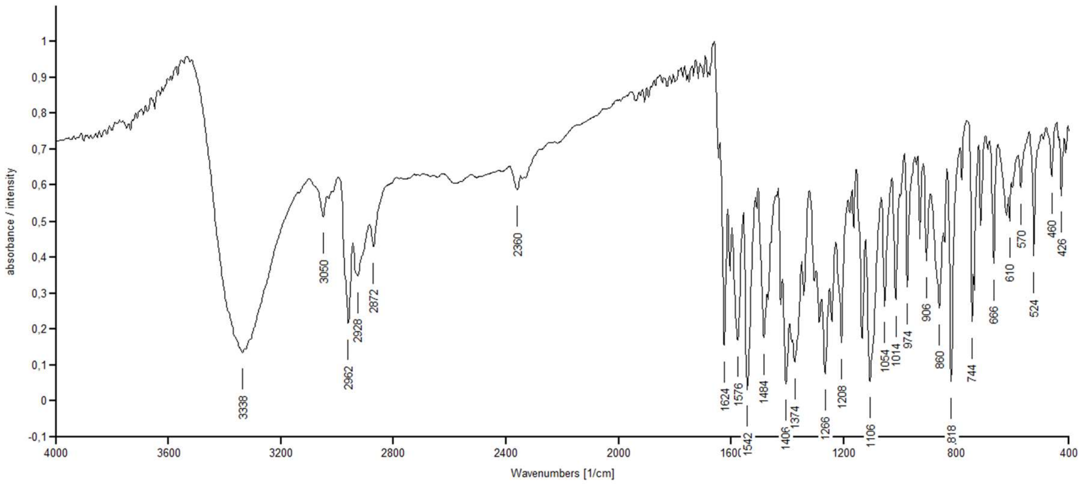

IR spectra for compound **1c**

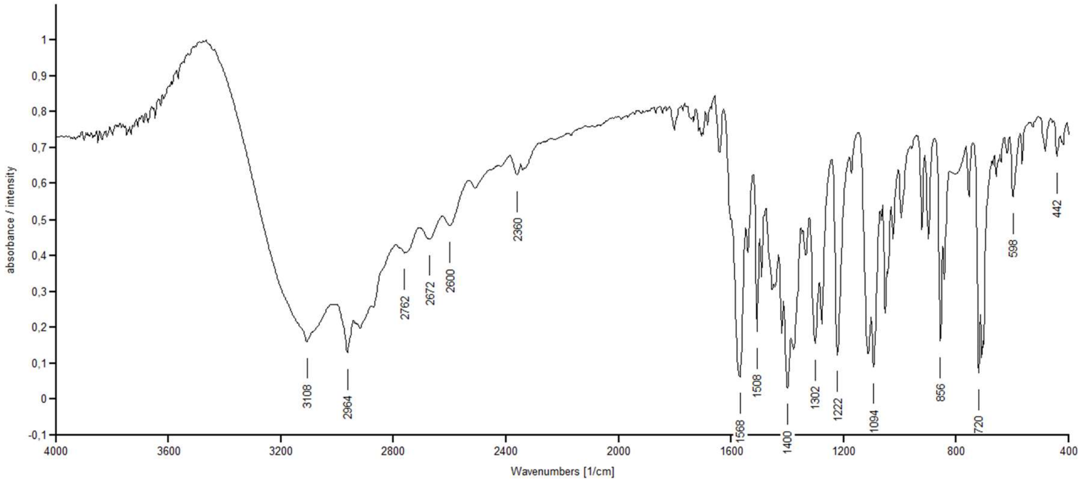

IR spectra for compound **1j**
