# Supplementary material for: Synthesis, Biological Evaluation and Docking Studies of Benzoxazoles Derived from Thymoquinone
Source: Molecules. 2018 Dec 12;23(12):3297. doi: 10.3390/molecules23123297 (PMC6321291; doi:10.3390/molecules23123297)
Supplement: Supplementary file 1 [file molecules-23-03297-s001.zip › molecules-399050- supplementary/molecules-399050-Mass spectra.pdf]

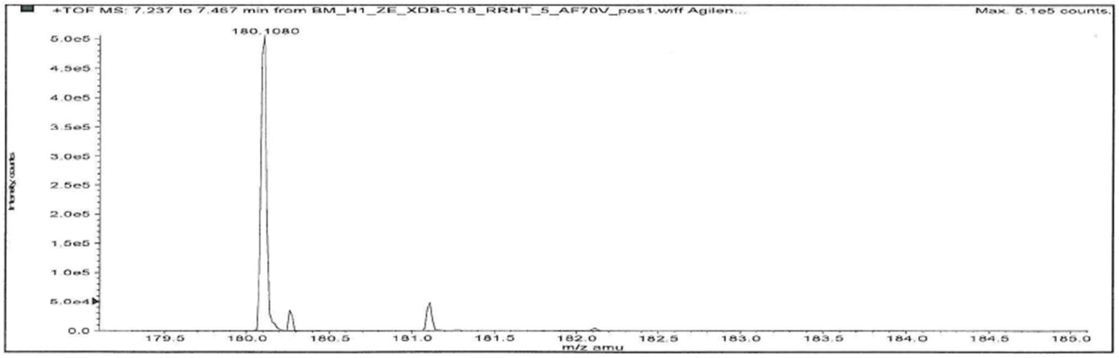

ESI-MS (*m*/*z*) spectra for compound **ATQ:** 180.10 [M + H]$^+$

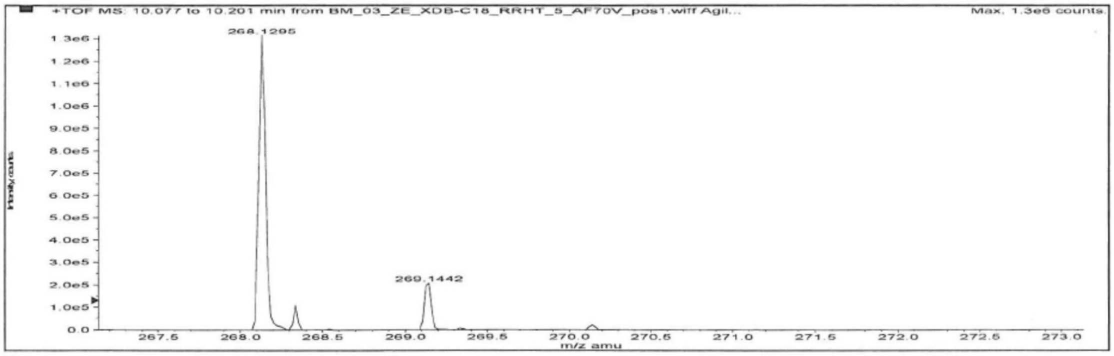

ESI-MS (*m*/*z*) spectra for compound **1a:** 268.13 [M + H]$^+$

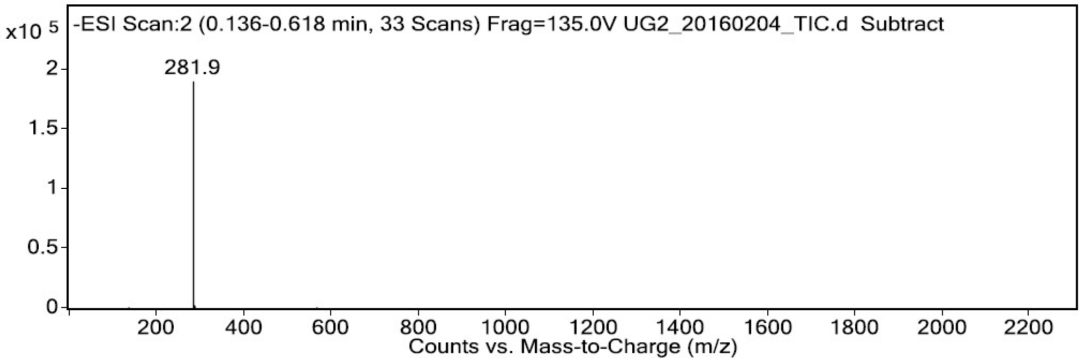

ESI-MS (*m*/*z*) spectra for compound **1b:** 284.1 [M + H]$^+$

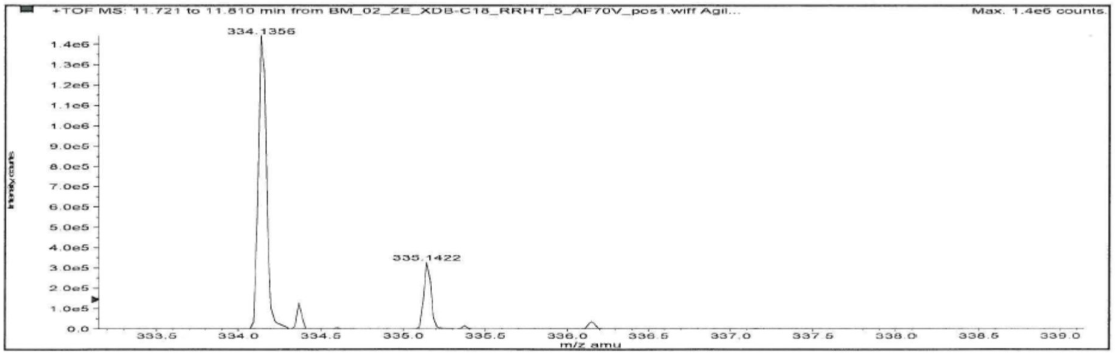

ESI-MS (*m*/*z*) spectra for compound **1c:** 334.14 [M + H]$^+$

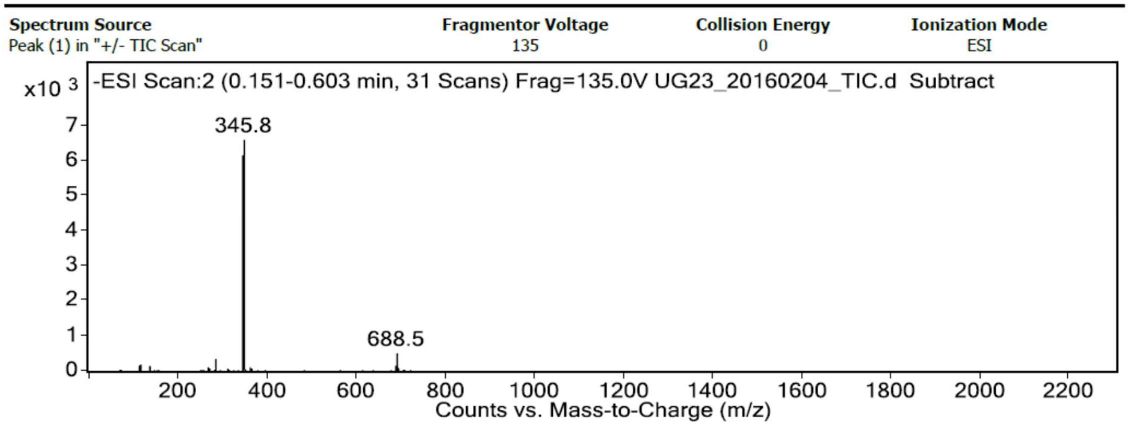

ESI-MS (*m*/*z*) spectra for compound **1d:** 346.0 [M + H]+

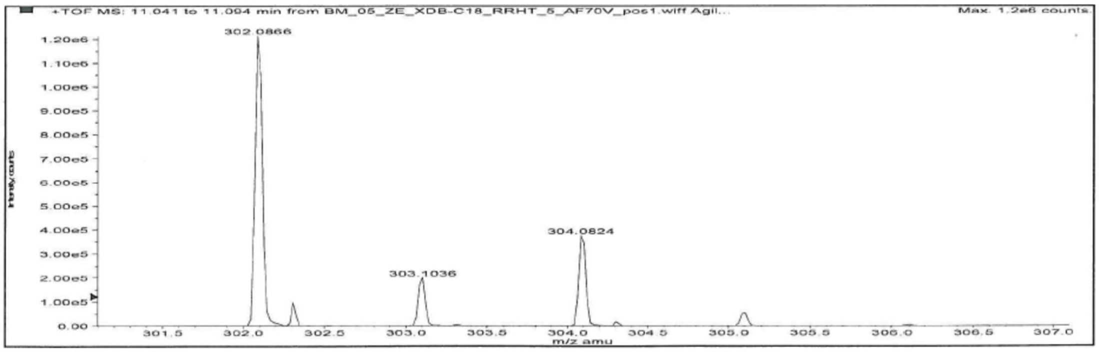

ESI-MS (*m*/*z*) spectra for compound **1e:** 302.09 [M + H]+

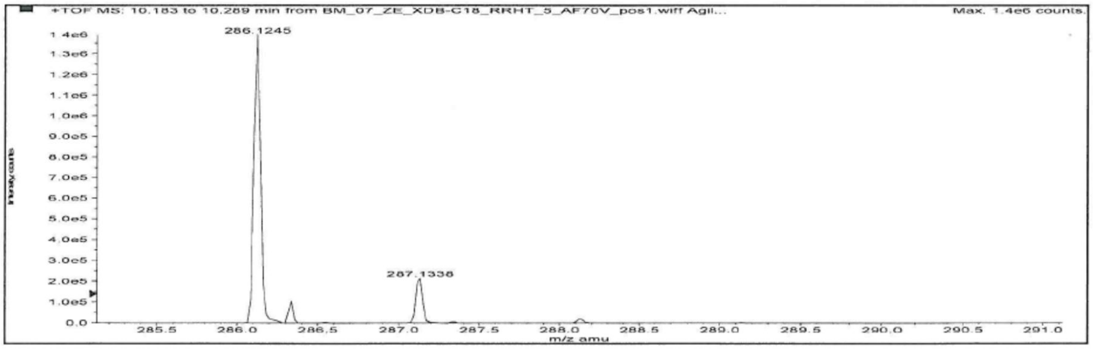

ESI-MS (*m*/*z*) spectra for compound **1f:** 286.12 [M + H]+

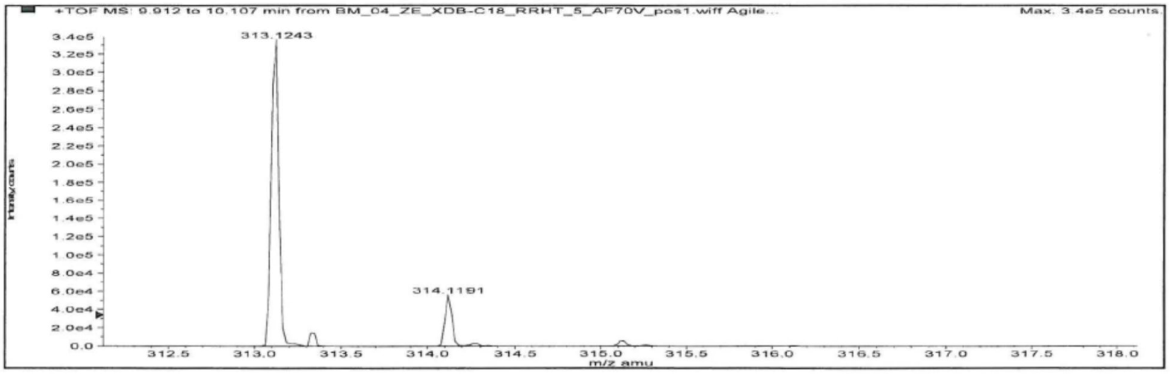

ESI-MS (*m*/*z*) spectra for compound **1g:** 313.12 [M + H]$^+$

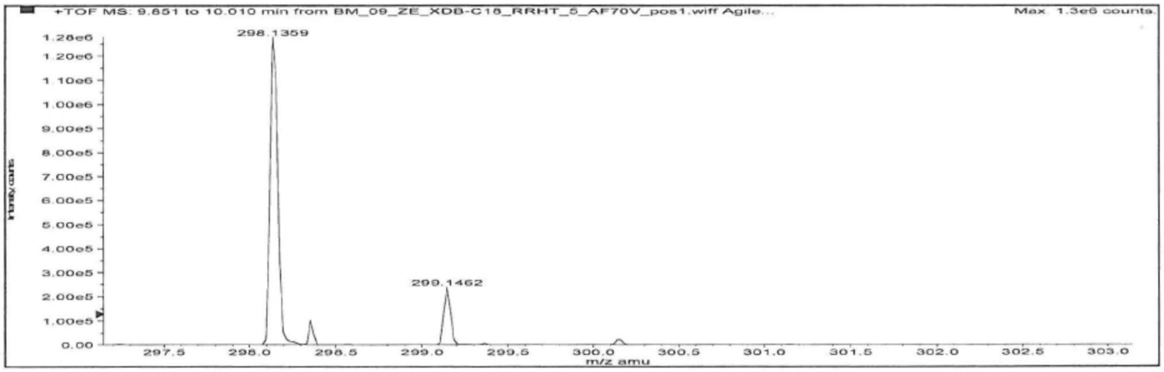

ESI-MS (*m*/*z*) spectra for compound **1h:** 298.14 [M + H]$^+$

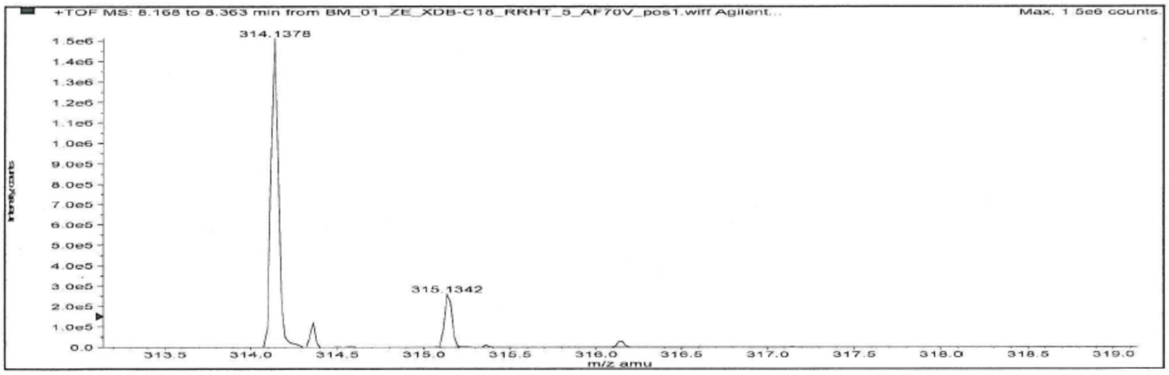

ESI-MS (*m*/*z*) spectra for compound **1i:** 314.14 [M + H]$^+$

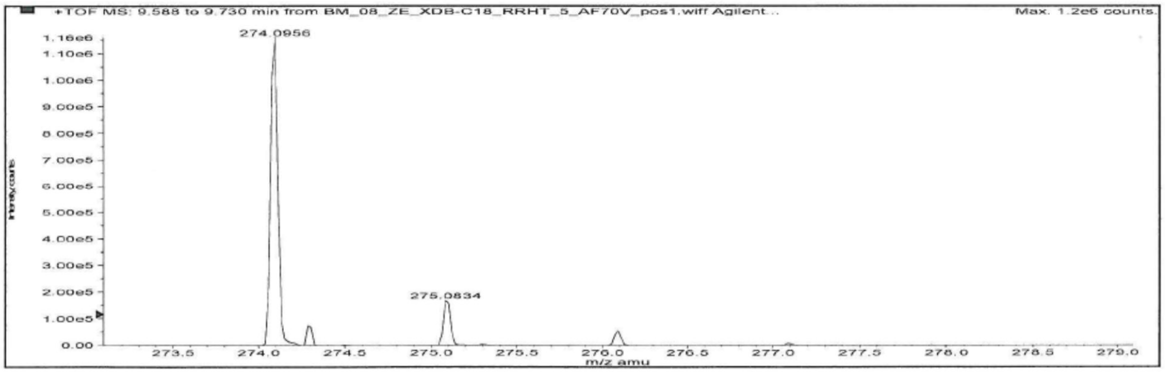

ESI-MS (*m*/*z*) spectra for compound **1j:** 274.09 $[M + H]^+$
