# Supplementary material for: Synthesis, Biological Evaluation and Docking Studies of Benzoxazoles Derived from Thymoquinone
Source: Molecules. 2018 Dec 12;23(12):3297. doi: 10.3390/molecules23123297 (PMC6321291; doi:10.3390/molecules23123297)
Supplement: Supplementary file 1 [file molecules-23-03297-s001.zip › molecules-399050- supplementary/molecules-399050-NMR spectra.pdf]

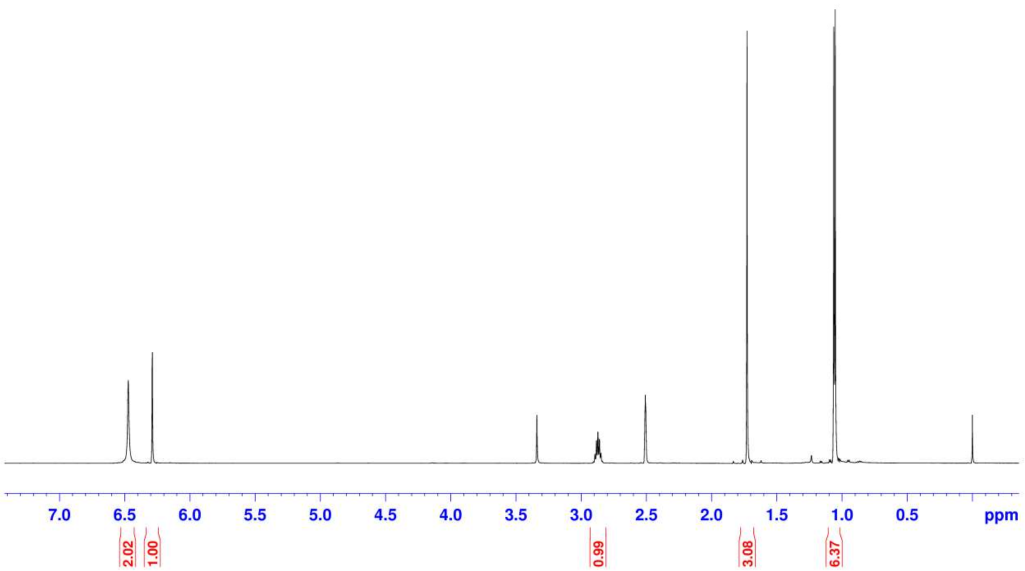

^{1}H NMR spectra for compound **ATQ**.

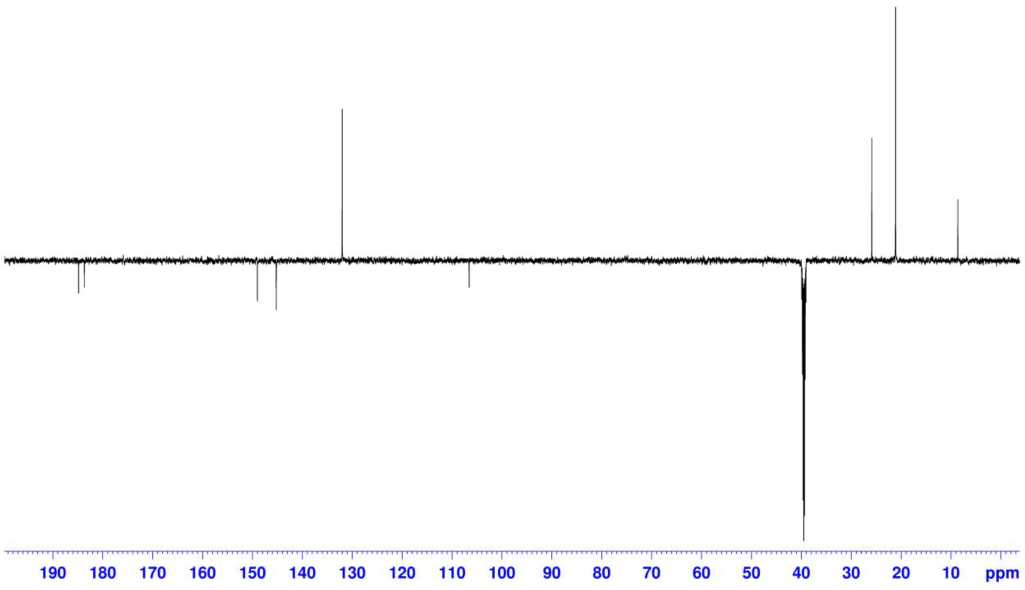

^{13}C APT NMR spectra for compound **ATQ**.

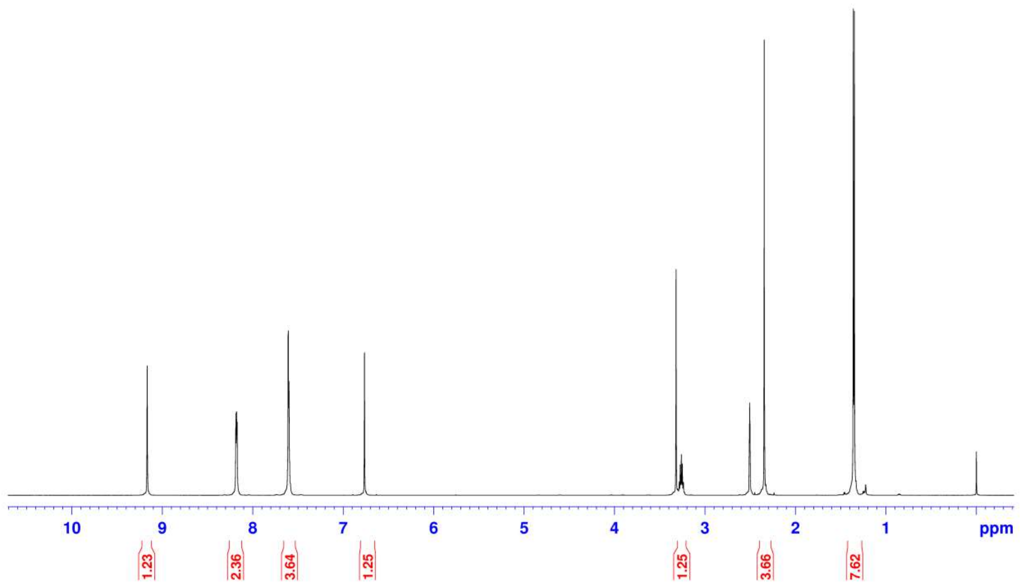

^{1}H NMR spectra for compound **1a**.

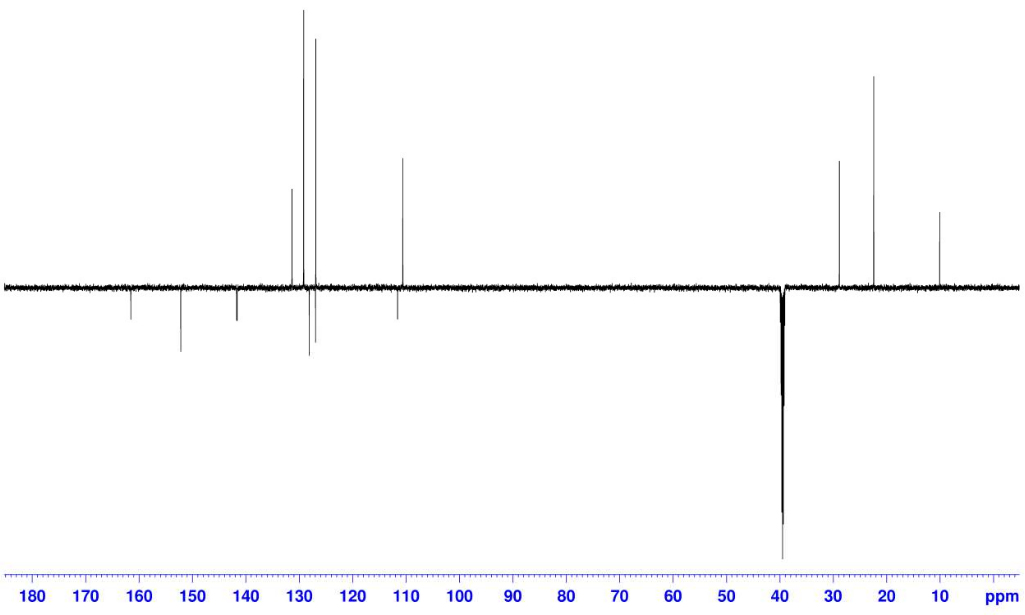

^{13}C APT NMR spectra for compound **1a**.

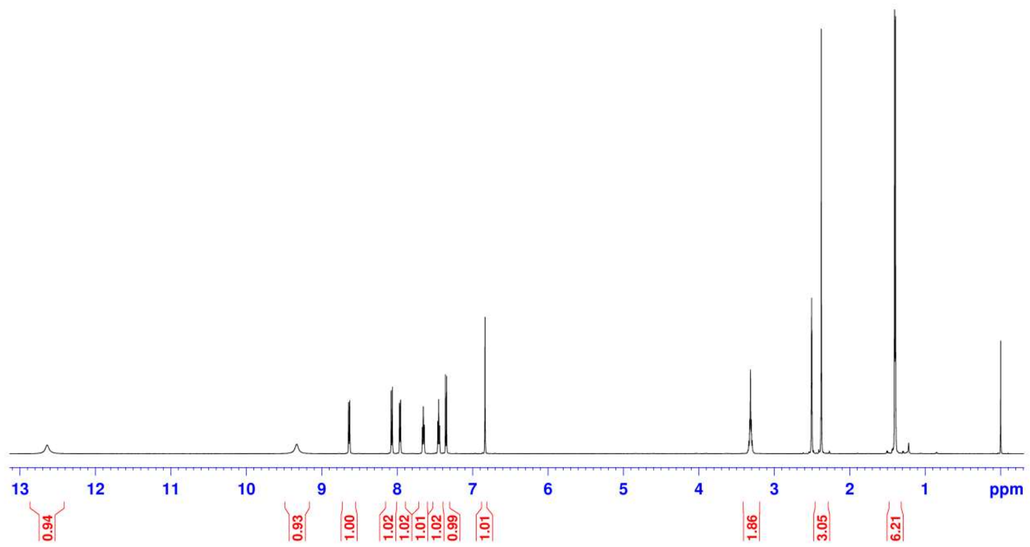

^{1}H NMR spectra for compound **1b**.

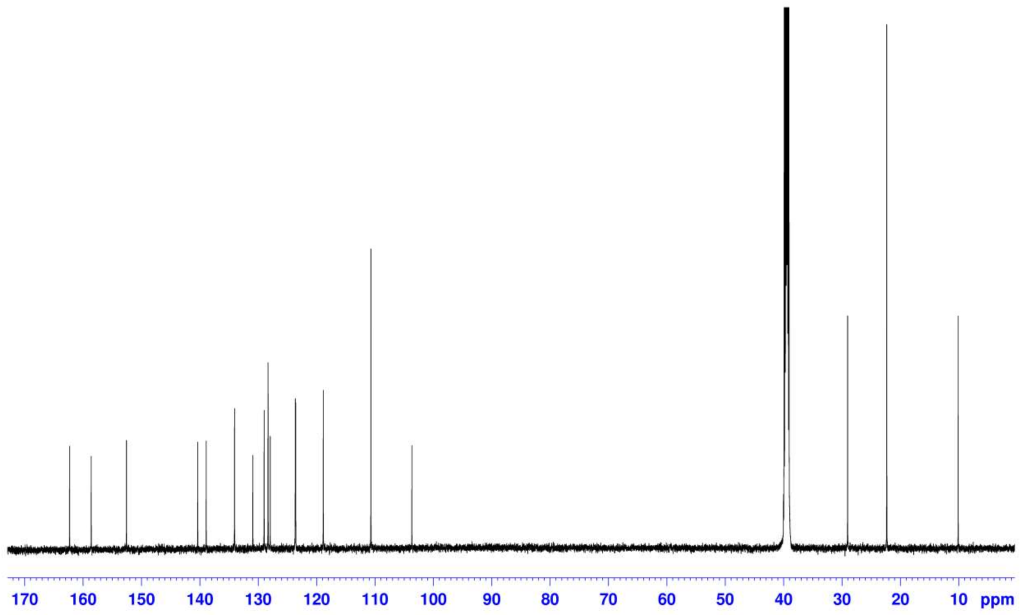

^{13}C {1H} NMR spectra for compound **1b**.

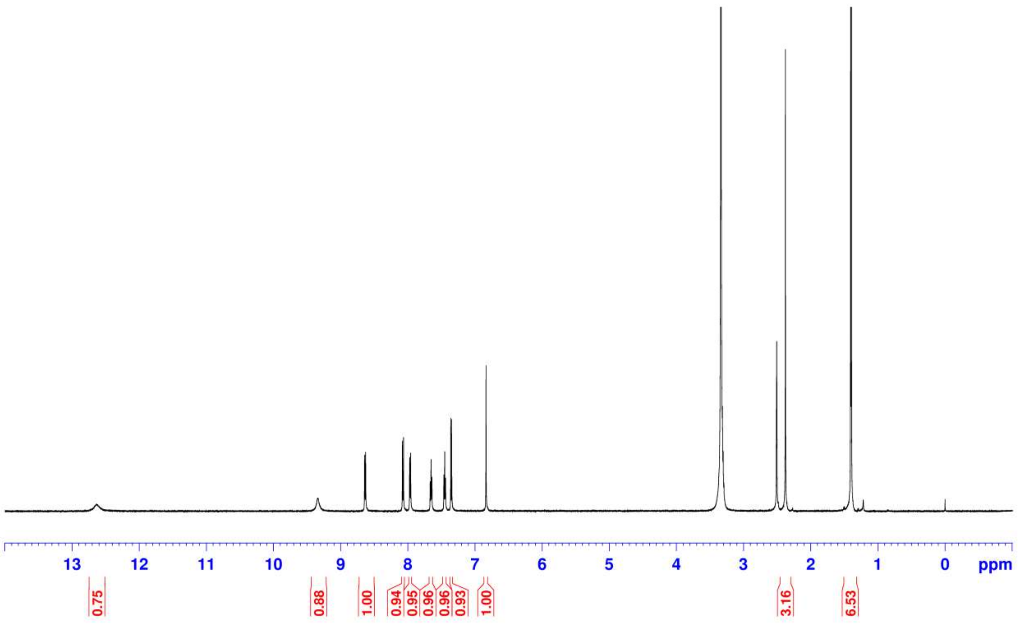

^{1}H NMR spectra for compound **1c**.

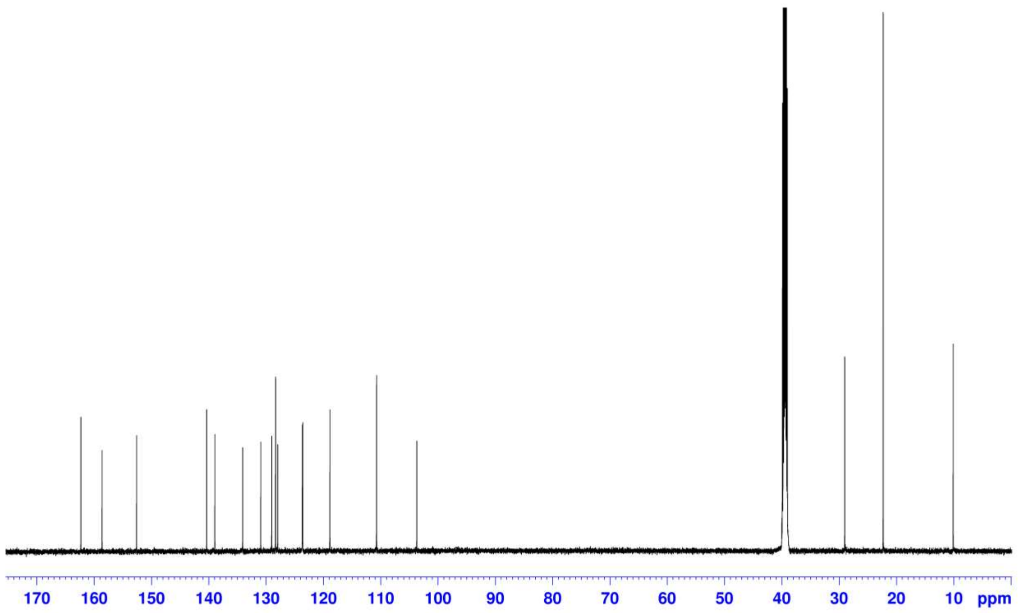

^{13}C {1H} NMR spectra for compound **1c**.

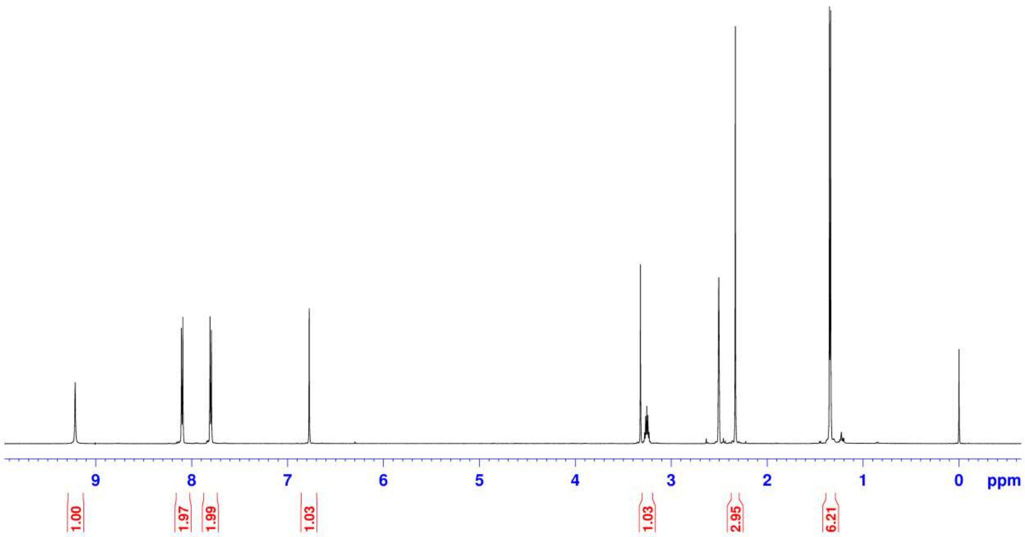

^{1}H NMR spectra for compound **1d**.

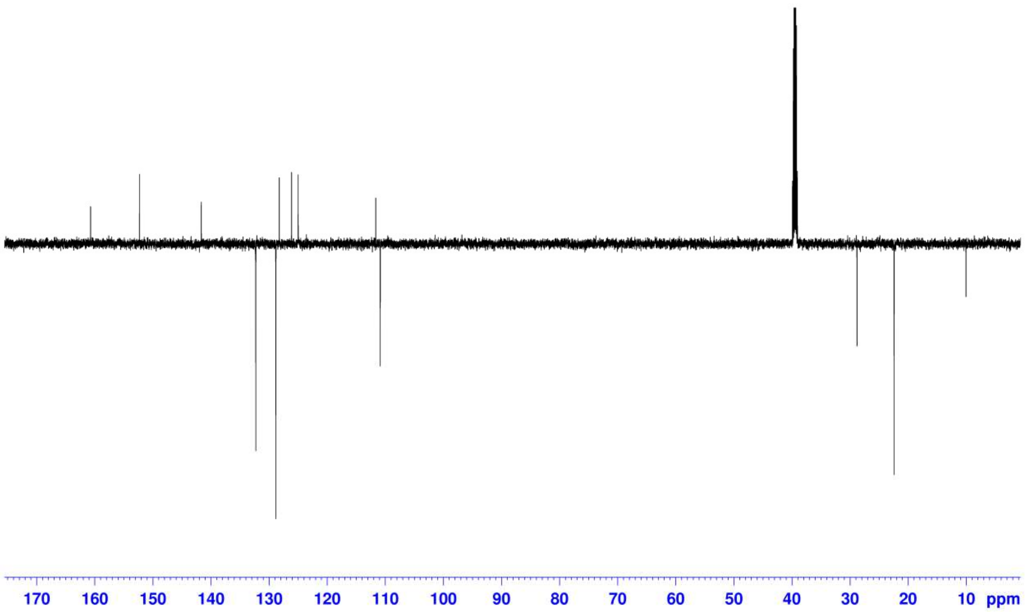

^{13}C APT NMR spectra for compound **1d**.

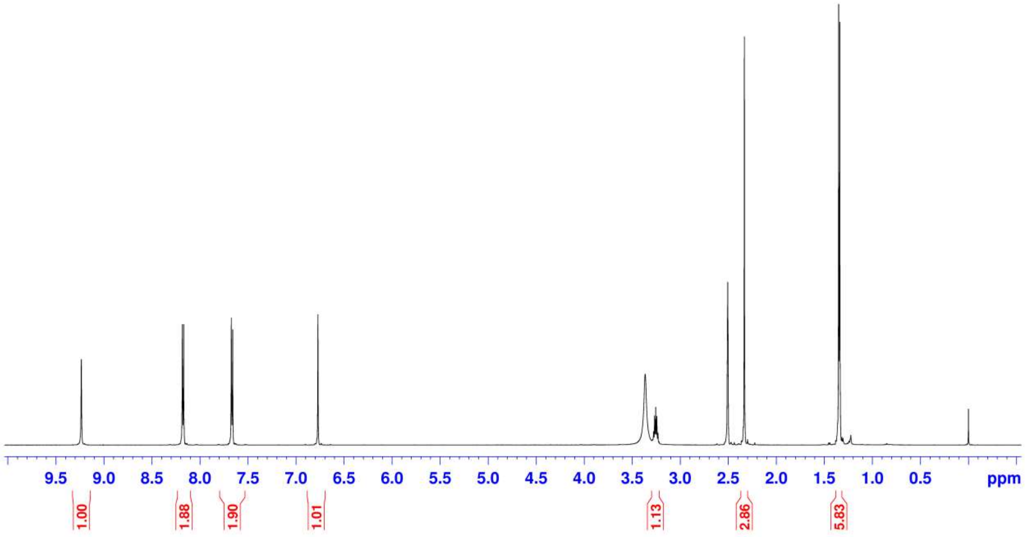

^{1}H NMR spectra for compound **1e**.

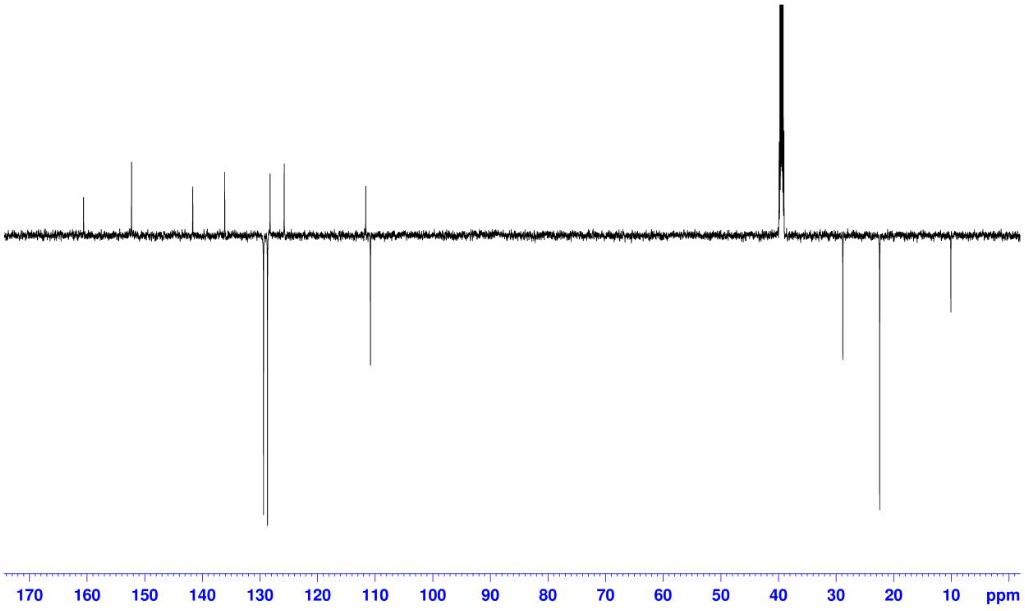

^{13}C APT NMR spectra for compound **1e**.

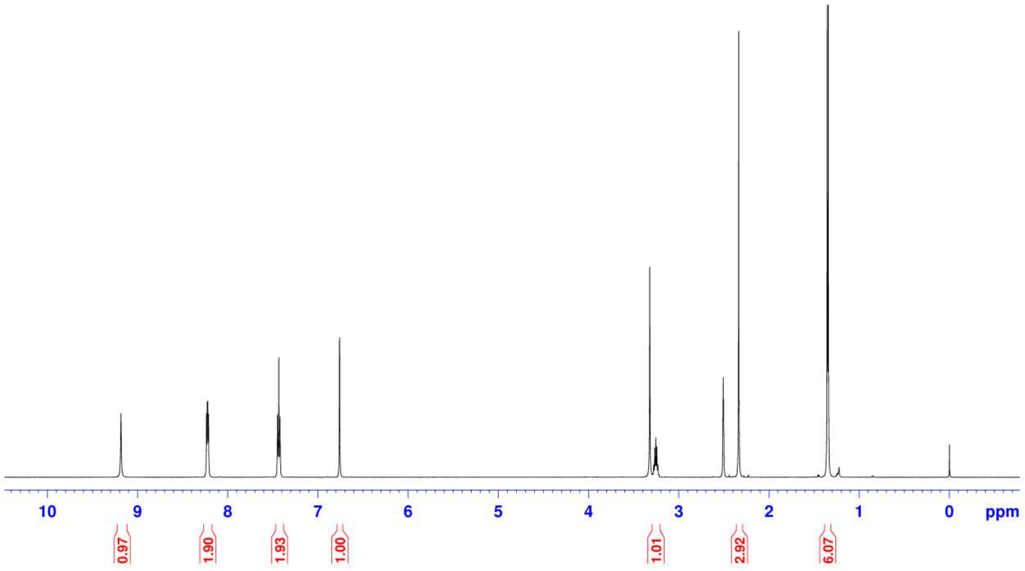

^{1}H NMR spectra for compound **1f**.

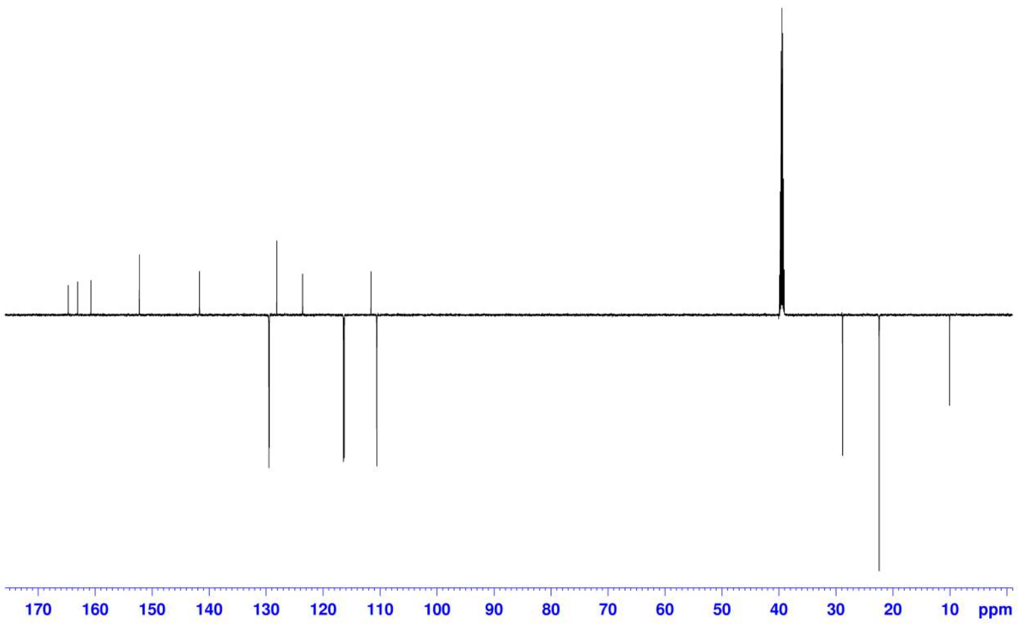

^{13}C APT NMR spectra for compound **1f**.

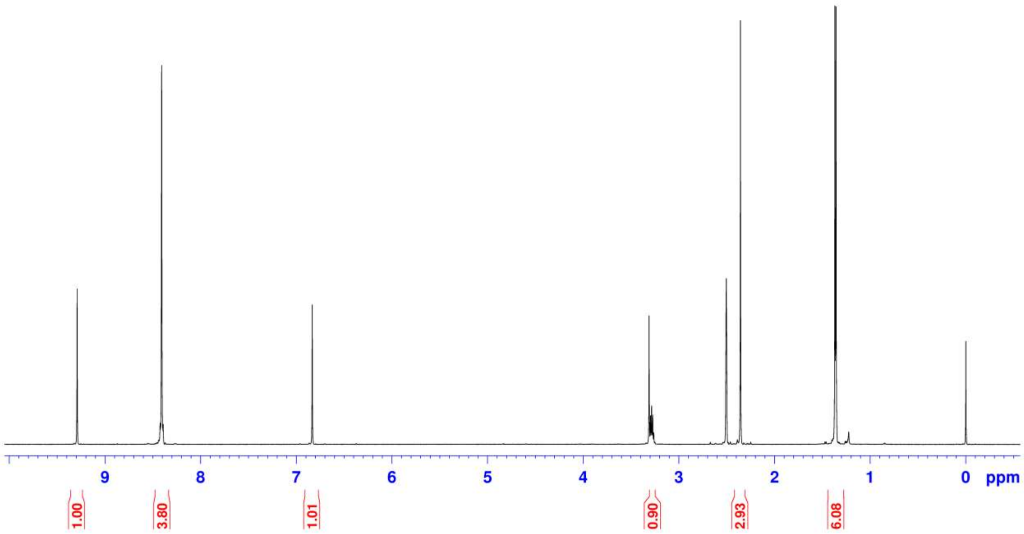

^{1}H NMR spectra for compound **1g**.

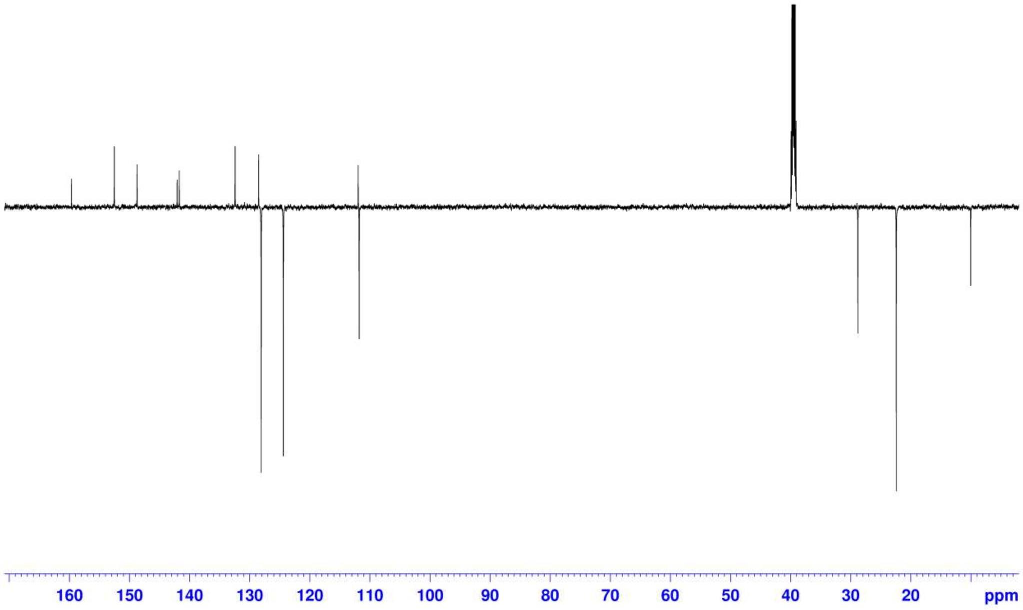

^{13}C APT NMR spectra for compound **1g**.

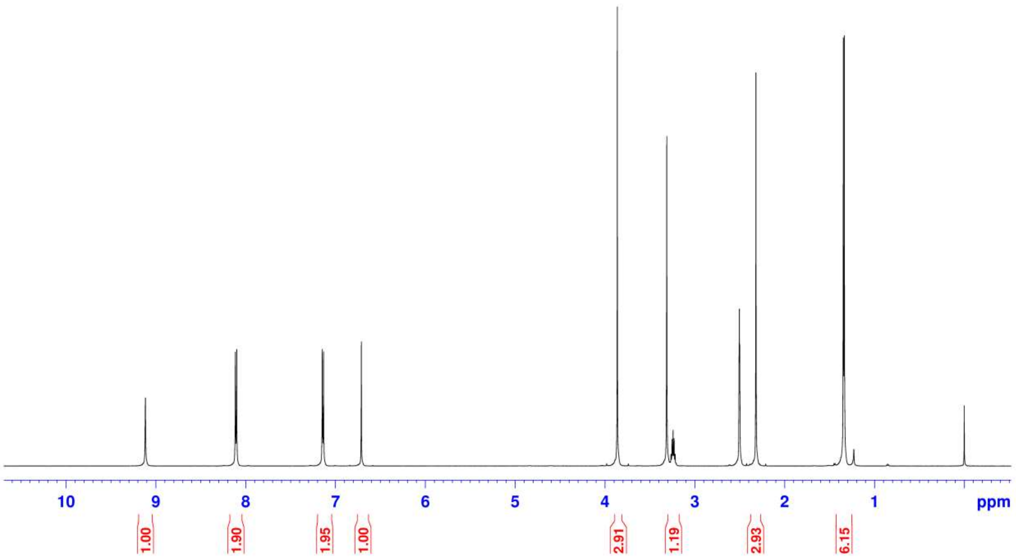

^{1}H NMR spectra for compound **1h**.

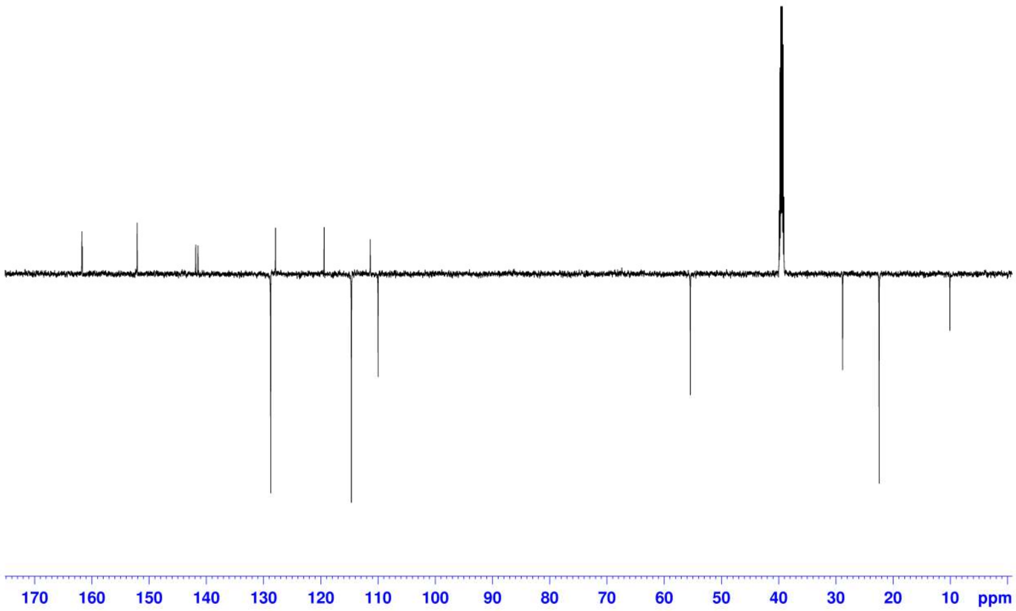

^{13}C APT NMR spectra for compound **1h**.

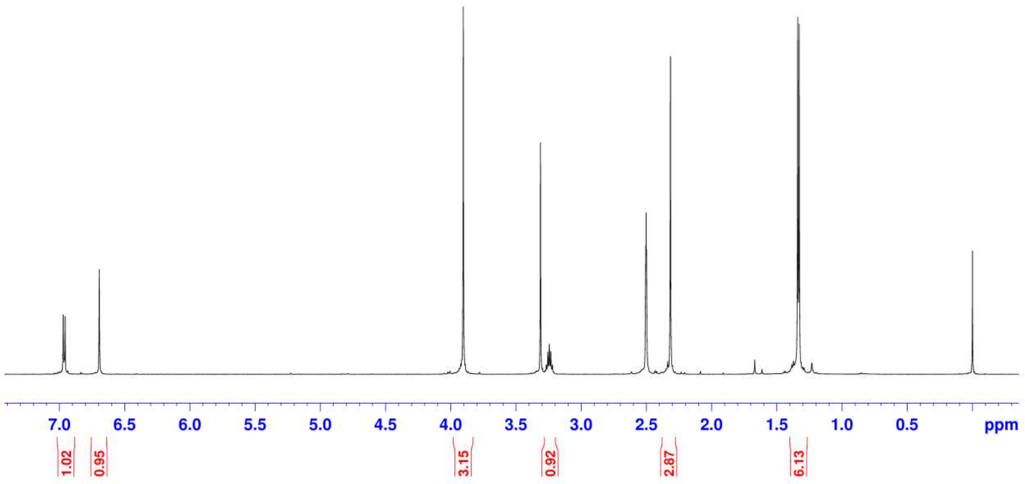

^{1}H NMR spectra for compound **1i**.

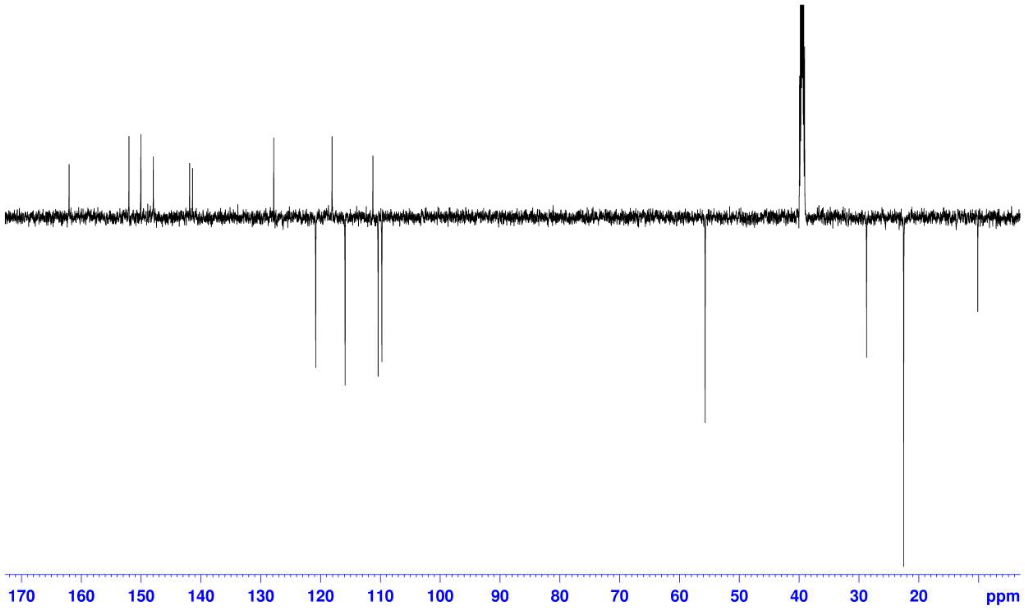

^{13}C APT NMR spectra for compound **1i**.

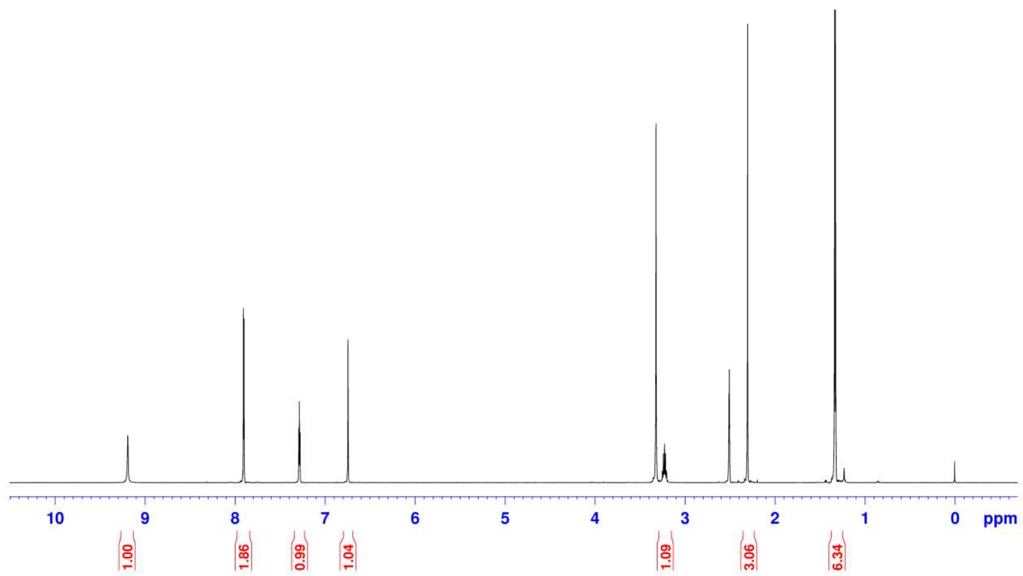

^{1}H NMR spectra for compound **1j**.

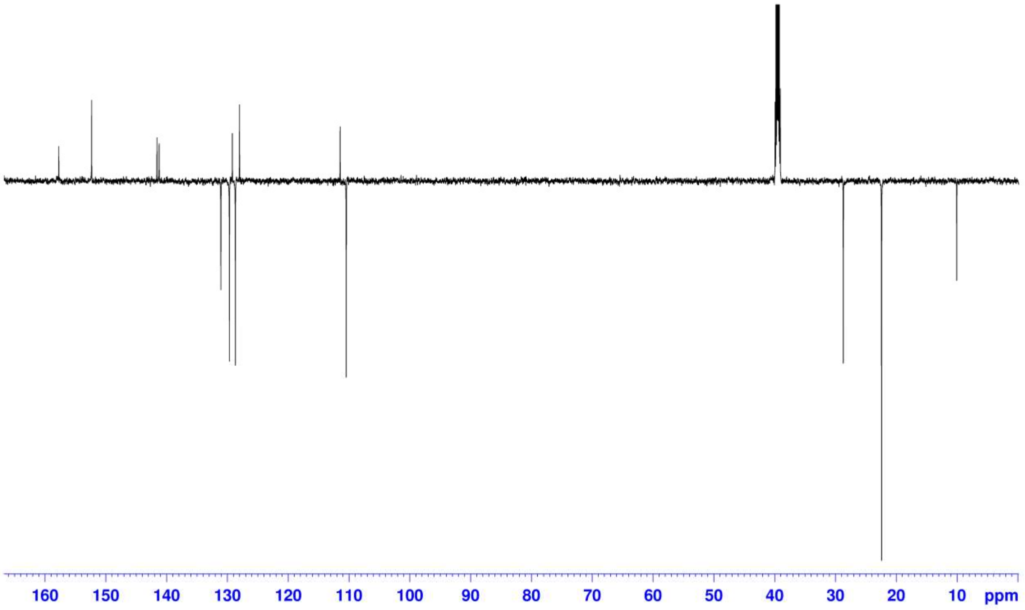

^{13}C APT NMR spectra for compound **1j**.
