# Supplementary material for: Synthesis, Biological Evaluation and Docking Studies of Benzoxazoles Derived from Thymoquinone
Source: Molecules. 2018 Dec 12;23(12):3297. doi: 10.3390/molecules23123297 (PMC6321291; doi:10.3390/molecules23123297)
Supplement: Supplementary file 1 [file molecules-23-03297-s001.zip › molecules-399050- supplementary/molecules-399050-Scheme 1, Table S1, Table S2.pdf]

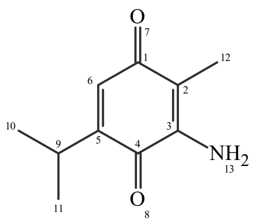

ATQ

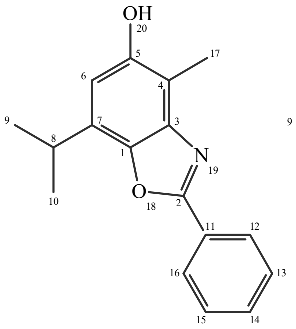

1a

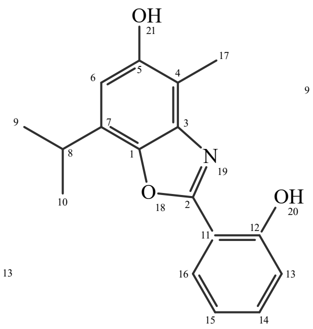

1b

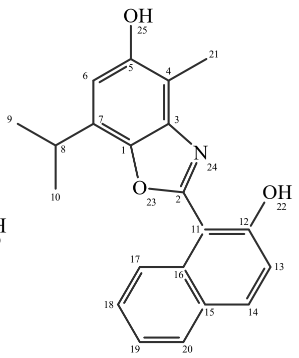

1c

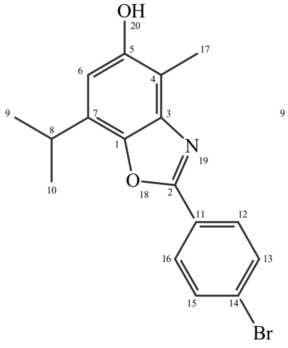

1d

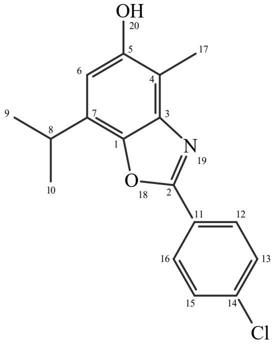

1e

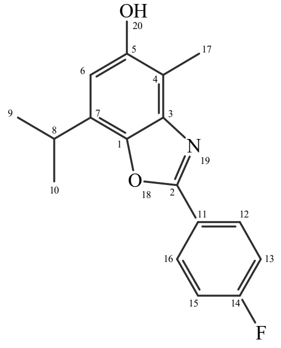

1f

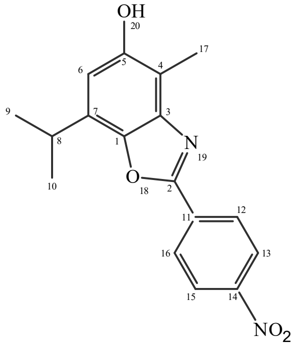

1g

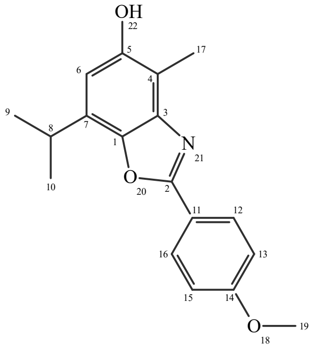

1h

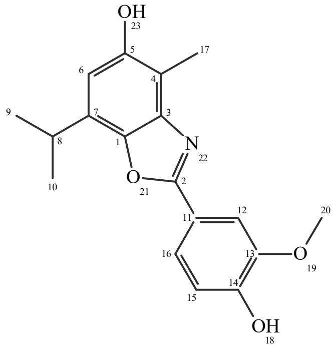

1i

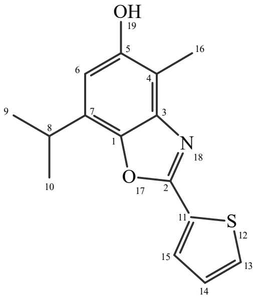

1j

Scheme S1. Numbering scheme for 3-aminothymoquinone (**ATQ**) and series of benzoxazoles (**1a–1j**).

Table S1. The crystal structure data of compound **1a.**

**Empirical Formula**	**$C_{17}H_{17}NO_2$**
M_w	267.31
T, K	150(2)
Crystal system	*monoclinic*
Space group	*P 2₁/n*
a, Å	7.7624 (2)
b, Å	18.3338 (5)
c, Å	10.0202 (3)
α, deg.	90
β, deg.	105.809 (3)
γ, deg.	90
V, $Å^3$	1372.08 (7)
Z	4
D_{calc}, g/cm^3	1.294
μ, mm^{-1}	0.676
F (000)	568
Crystal size, mm	0.60 × 0.60 × 0.40
Color	colorless
Data collected/unique	5041/2687
R_{int}	0.0178
Restraints/parameters	0/186
S	1.021
R_1, wR_2 [I > 2σ(I)]	0.0382/0.1026
R_1, wR_2 (all data)	0.0416/0.1056
Larg. diff. peak/hole (e·$Å^{-3}$)	0.23/−0.19

Table S2. Mobile phases used in purification of compounds **1a-1j.**

Compound	Mobile Phase
**1a**	*n*-hexane: Dichloromethane (DCM): Tetrahydrofuran (THF) = 50:50:1.4, gradually increasing quantity of THF until final concentration of 10% THF in combination
**1b**	*n*-hexane, then *n*-hexane: Diethyl ether (DEE) = 30:5
**1c**	*n*-hexane: DCM = 1:1, then *n*-hexane: DCM = 8:12, and at the end DCM 100%
**1d**	*n*-hexane, then *n*-hexane: THF = 12:1
**1e**	*n*-hexane: DCM: THF = 50:50:4
**1f**	*n*-hexane: DCM: THF = 50:50:2, and after that content of n-hexane and DHM are decreased while content of THF is increased in ratios 30:30:3 and 20:20:4
**1g**	*n*-hexane, after that DEE is gradually added with increasing ratios n-hexane: DEE = 30:1, 30:2, 30:3, 30:5, 30:10 and 30:15
**1h**	*n*-hexane: DCM: THF = 50:50:3, after that content of THF is increased 50:50:4, 50:50:5, 50:50:8, 50:50:10, 50:50:11 and 50:50:12
**1i**	*n*-hexane: DEE = 2:1
**1j**	*n*-hexane: DCM: THF = 50:50:2.5
